# Evolving neoantigen profiles in colorectal cancers with DNA repair defects

**DOI:** 10.1186/s13073-019-0654-6

**Published:** 2019-06-28

**Authors:** Giuseppe Rospo, Annalisa Lorenzato, Nabil Amirouchene-Angelozzi, Alessandro Magrì, Carlotta Cancelliere, Giorgio Corti, Carola Negrino, Vito Amodio, Monica Montone, Alice Bartolini, Ludovic Barault, Luca Novara, Claudio Isella, Enzo Medico, Andrea Bertotti, Livio Trusolino, Giovanni Germano, Federica Di Nicolantonio, Alberto Bardelli

**Affiliations:** 1 Candiolo Cancer Institute, FPO-IRCCS, 10060 Candiolo (TO), Italy; 2 Department of Oncology, University of Turin, 10060 Candiolo (TO), Italy; 3 FIRC Institute of Molecular Oncology (IFOM), 20139 Milan, Italy

**Keywords:** Colorectal cancer, Neoantigen, DNA repair, Immune response, Mutational signature

## Abstract

**Background:**

Neoantigens that arise as a consequence of tumor-specific mutations can be recognized by T lymphocytes leading to effective immune surveillance. In colorectal cancer (CRC) and other tumor types, a high number of neoantigens is associated with patient response to immune therapies. The molecular processes governing the generation of neoantigens and their turnover in cancer cells are poorly understood. We exploited CRC as a model system to understand how alterations in DNA repair pathways modulate neoantigen profiles over time.

**Methods:**

We performed whole exome sequencing (WES) and RNA sequencing (RNAseq) in CRC cell lines, in vitro and in vivo, and in CRC patient-derived xenografts (PDXs) to track longitudinally genomic profiles, clonal evolution, mutational signatures, and predicted neoantigens.

**Results:**

The majority of CRC models showed remarkably stable mutational and neoantigen profiles; however, those carrying defects in DNA repair genes continuously diversified. Rapidly evolving and evolutionary stable CRCs displayed characteristic genomic signatures and transcriptional profiles. Downregulation of molecules implicated in antigen presentation occurred selectively in highly mutated and rapidly evolving CRC.

**Conclusions:**

These results indicate that CRCs carrying alterations in DNA repair pathways display dynamic neoantigen patterns that fluctuate over time. We define CRC subsets characterized by slow and fast evolvability and link this phenotype to downregulation of antigen-presenting cellular mechanisms. Longitudinal monitoring of the neoantigen landscape could be relevant in the context of precision medicine.

**Electronic supplementary material:**

The online version of this article (10.1186/s13073-019-0654-6) contains supplementary material, which is available to authorized users.

## Background

Anticancer therapies based on immune-checkpoint blockade are often remarkably effective but benefit only a minor fraction of cancer patients [1]. Several biomarkers of response and resistance to immune modulators have been proposed [2, 3]. Among these, the overall mutational burden (number of somatic variants per megabase (Mb)) and the number of predicted neoantigens were highlighted in multiple studies [4–6]. The predictive values of mutational and antigen burdens are still being evaluated in clinical settings. Both parameters are presently assessed on DNA extracted from individual tissue samples and are typically measured only once in the clinical history of each patient. Alterations in DNA repair pathways, including mutations or promoter hypermethylation of mismatch repair (MMR) effectors (*MLH1*, *MSH2*, etc.) or DNA polymerases (polymerase ε and δ) [7], are known to increase the mutational burden and the neoantigen profiles of cancers [8]. Whether, and to what extent, neoantigen profiles evolve over time as a result of the inherent genomic instability of individual tumors is largely unknown. We recently reported that in mouse models, inactivation of DNA mismatch repair increases the mutational burden and leads to dynamic mutational profiles resulting in effective cancer immune response [9]. Here we exploit CRCs as a model system to understand whether mutational burden and neoantigen profile of human tumors evolve over time as a result of their distinctive genomic landscapes.

## Methods

### CRC cell lines

The source of each cell line is reported in Table 1. All cell lines were maintained in their original culturing conditions according to supplier guidelines. Cells were ordinarily supplemented with FBS 10%, 2 mM l-glutamine, and antibiotics (100 U/mL penicillin and 100 mg/mL streptomycin) and grown in a 37 °C and 5% CO_2_ air incubator. To study the evolution of cell populations, cell lines were not cloned prior to the experiment or at any subsequent time point. Cell lines were thawed in a 10-cm dish. After thaw recovery, each cell line was screened for the absence of mycoplasma contamination and checked for its identity, referred below as quality control (QC). To preserve heterogeneity, upon thawing, individual lines were expanded to at least 10^8^ cells. At this point for each model, cells were counted, and the percentage of alive/dead cells was calculated. At the beginning of the experiment (T0), 4 × 10^7^ live cells were distributed as follows: (A) 2 × 10^6^ cells were re-plated in a 10-cm dish for in vitro propagation, (B) 3 × 10^7^ cells were used for in vivo experiments, (C) 2 × 10^6^ cells were frozen, and (D) 3 pellets (2 × 10^6^ cells each) were frozen for DNA, RNA, and protein extraction. Cells plated as in (A) were kept in culture changing medium twice a week and dividing them at a constant splitting rate, determined before initiating the experiment. In details, splitting was performed before full confluency was achieved. The number of cells that were split and the number of passages and days of culture were recorded for each cell model to calculate the doubling time. During in vitro culture, cell populations were collected at the following pre-determined time points: 30 days (T30), 60 days (T60) and 90 days (T90) from T0. At each time point, a fraction of the cells were put aside (note that this did not affect the rate of passaging described below) and pellets (2 × 10^6^ each) were collected for DNA, RNA, and protein extraction. QC was repeated at each time point.

**Table 1:** Molecular, functional characteristics and source of origin of the indicated cell lines

Sample	Microsatellite status	Altered MSI markers	Genome evolvability	Split ratio	In vitro doubling time	Growth rate	Source
C10	Stable	–	Stable	0.34	2.33	0.30	ECACC
C106	Stable	–	Stable	0.35	2.50	0.28	ECACC
C125PM	Stable	–	Stable	0.34	2.37	0.29	ECACC
C32	Stable	–	Stable	0.18	1.54	0.45	ECACC
C70	Stable	–	Stable	0.4	2.76	0.25	ECACC
C75	Stable	–	Stable	0.36	2.46	0.28	ECACC
C99	Stable	–	NA	NA	NA	NA	ECACC
CACO2	Stable	–	Stable	0.26	1.83	0.38	ATCC
CAR1	Stable	–	Stable	0.36	2.47	0.28	JCRB
CCK81	Unstable	bat26-nr21-bat25-mono27-nr24	Evolving	0.34	2.53	0.27	RIKEN
CL14	Stable	–	NA	NA	NA	NA	DSMZ
COCM1	Stable	–	Evolving	0.3	2.34	0.30	JCRB
COGA1	Unstable	bat26-mono27-nr24	NA	NA	NA	NA	Dr. Huber^a^
COGA2	Stable	–	Stable	0.32	2.22	0.31	Dr. Huber^a^
COGA5	Stable	–	Stable	0.20	1.69	0.41	Dr. Huber^a^
COGA8	Stable	–	Stable	0.22	1.67	0.41	Dr. Huber^a^
COLO201	Stable	–	NA	NA	NA	NA	ATCC
COLO94H	Stable	–	Stable	0.45	2.81	0.25	CLS
DIFI	Stable	–	NA	NA	NA	NA	Dr. Baselga^b^
DLD1	Unstable	bat26-nr21-bat25-mono27-nr24	Evolving	0.07	0.98	0.71	NCI60
HCA24	Stable	–	Evolving	0.33	2.22	0.31	ECACC
HCA46	Stable	–	Stable	0.4	2.41	0.29	ECACC
HCC2998	Stable	–	Stable	0.34	2.33	0.30	NCI60
HDC114	Stable	–	Evolving	0.23	1.69	0.41	DKFZ^c^
HDC142	Stable	–	Evolving	0.35	2.42	0.29	DKFZ^c^
HDC82	Stable	–	NA	NA	NA	NA	DKFZ^c^
HRA16	Stable	–	NA	NA	NA	NA	ECACC
HROC24	Unstable	bat26-nr21-bat25-mono27-nr24	Evolving	0.16	1.23	0.56	Dr. Linnebacher^d^
HROC32	Stable	–	Stable	0.62	6.07	0.11	Dr. Linnebacher^d^
HROC334	Stable	–	Stable	0.45	2.83	0.24	Dr. Linnebacher^d^
HROC39	Stable	–	Stable	0.5	3.92	0.18	Dr. Linnebacher^d^
HROC69	Stable	–	Stable	0.33	2.26	0.31	Dr. Linnebacher^d^
HT115	Stable	–	Evolving	0.24	1.78	0.39	ECACC
HT29	Stable	–	Stable	0.16	1.38	0.50	NCI60
HT55	Stable	–	Stable	0.33	2.21	0.31	ECACC
LIM1215	Unstable	bat26-nr21-bat25-mono27-nr24	Evolving	0.15	1.24	0.56	Dr. Whitehead^e^
LIM2099	Stable	–	Stable	0.33	2.26	0.31	Dr. Whitehead^e^
LOVO	Unstable	nr21-bat25-mono27-nr24	NA	NA	NA	NA	ATCC
LS180	Unstable	bat26-nr21-bat25-mono27-nr24	Evolving	0.25	1.90	0.37	ATCC
LS411N	Unstable	bat26-nr21-bat25-mono27-nr24	Evolving	0.32	2.29	0.30	ATCC
MDST8	Stable	–	Stable	0.15	1.31	0.53	ECACC
NCIH716	Stable	–	NA	NA	NA	NA	ATCC
OUMS23	Stable	–	Stable	0.26	1.70	0.41	JCRB
OXCO3	Stable	–	Stable	0.23	1.76	0.39	Dr. Cerundolo^f^
RW7213	Stable	–	Stable	0.4	2.60	0.27	Dr. Arango^g^
SNU1040	Unstable	bat26-nr21-bat25-mono27-nr24	Evolving	0.54	4.14	0.17	KCLB
SNU1181	Stable	–	Stable	0.62	4.65	0.15	KCLB
SNU1235	Stable	–	Evolving	0.35	2.33	0.30	KCLB
SNU1411	Stable	–	Evolving	0.44	2.76	0.25	KCLB
SNU1460	Stable	–	NA	NA	NA	NA	KCLB
SNU1684	Unstable	bat26-nr21-bat25-mono27-nr24	NA	NA	NA	NA	KCLB
SNU175	Unstable	bat26-nr21-bat25-mono27	NA	NA	NA	NA	KCLB
SNU283	Stable	–	NA	NA	NA	NA	KCLB
SNU479	Stable	–	NA	NA	NA	NA	KCLB
SNU81	Stable	–	Evolving	0.42	2.33	0.30	KCLB
SNU977	Stable	–	Stable	0.42	2.71	0.26	KCLB
SNUC1	Stable	–	NA	NA	NA	NA	KCLB
SW1417	Stable	–	NA	NA	NA	NA	ATCC
SW1463	Stable	–	NA	NA	NA	NA	ATCC
SW480	Stable	–	Stable	0.18	1.64	0.42	ATCC
SW837	Stable	–	Stable	0.27	2.07	0.34	ATCC
V411	Stable	–	Stable	0.22	1.91	0.36	Dr. Markovitz^h^
V481	Unstable	bat26-nr21-bat25-mono27-nr24	NA	NA	NA	NA	Dr. Markovitz^h^
WIDR	Stable	–	NA	NA	NA	NA	Dr. Bernards^i^

Non-commercial cell lines were provided by (a) Dr. L. A. Huber, Cell Biology/Biocenter, Medical University of Innsbruck, Innsbruck, Austria; (b) Dr. Baselga, Chairman & Professor of Medicine, Vall d’Hebron Institute of Oncology (V.H.I.O.), Vall d’ Hebron University Hospital, Barcelona, Spain; (c) Dr. M. Schawb, Division of Tumour Genetics - B030 German Cancer Research Center (DKFZ), Heidelberg, Germany; (d) Dr. M. Linnebacher, Division of Molecular Oncology and Immunotherapy, Department of General Surgery, University of Rostock, Rostock, Germany; (e) Dr. R.H. Whitehead, Depts of Medicine, Cell and Developmental Biology and Cancer Biology, Vanderbilt University, Nashville, USA; (f) Dr. V. Cerundolo, Nuffield Dept of Clinical Medicine, John Radcliffe Hospital, Oxford, UK; (g) Dr. D. Arango, Group of Molecular Oncology, Nanomedicine Research Program, Molecular Biology and Biochemistry Research Center, CIBBIM Nanomedicine, Vall d’Hebron, Barcelona, Spain; (h) Dr. S. Markovitz, Case Comprehensive Cancer Center, Division of Hematology-Oncology, Department of Medicine, Case Western Reserve University, Cleveland, USA; (i) Dr. R. Bernards, Division of Molecular Carcinogenesis B7, Netherlands Cancer Institute, Amsterdam, The Netherlands.

### Cell quality control (QC)

Cells were screened for the absence of mycoplasma contamination using the Venor®GeM Classic kit (Minerva Biolabs). The identity of each cell line was checked before starting each experiment and after every genomic DNA extraction by PowerPlex® 16 HS System (Promega), through Short Tandem Repeats (STR) at 16 different loci (D5S818, D13S317, D7S820, D16S539, D21S11, vWA, TH01, TPOX, CSF1PO, D18S51, D3S1358, D8S1179, FGA, Penta D, Penta E, and amelogenin). Amplicons from multiplex PCRs were separated by capillary electrophoresis (3730 DNA Analyzer, Applied Biosystems) and analyzed using GeneMapper v 3.7 software (Life Technologies).

### Microsatellite instability (MSI) status

The MSI status was assessed with the MSI Analysis System kit (Promega). The analysis requires a multiplex amplification of seven markers including five mononucleotide repeat markers (BAT-25, BAT-26, NR-21, NR-24, and MONO-27) and two pentanucleotide repeat markers (Penta C and Penta D). The products were analyzed by capillary electrophoresis in a single injection (3730 DNA Analyzer, ABI capillary electrophoresis system (Applied Biosystems). Then, the results were analyzed using GeneMapper V5.0 software.

### DNA extraction and exome sequencing

Genomic DNA (gDNA) was extracted from CRC cell lines, xenografts, and PDXs using Maxwell® RSC Blood DNA kit (AS1400, Promega). DNA was sent to IntegraGen SA (Evry, France) that performed library preparation, exome capture, sequencing, and data demultiplexing. Final DNA libraries were pair-end sequenced on Illumina HiSeq4000 as paired-end 100 bp reads.

### Mutational analysis in cell lines

When cell lines were passaged in mice or when analyzing patient-derived xenografts, Fastq files were first processed with Xenome [10] to remove reads of mouse origin. Reads files were aligned to the human reference hg38 using BWA-mem algorithm [11], and then the “rmdup” samtools command was used to remove PCR duplicates [12]. On the resulting aligned files, we observed a median depth of 138x with 98% of the targeted region covered by at least one read. Bioinformatic modules previously developed [9, 13] by our laboratory were used to identify single nucleotide variants (SNVs) and indels. The mutational characterization of the 64 cell lines at time point 0 was assessed by calling the alterations against the hg38 reference annotation. Then, a series of filters were used to remove germline variants and artifacts: alleles supported by only reads with the same strand, excluding start and end read positions from the count, were discarded; variants called with allelic frequency lower than 10% as well a *p* value greater than 0.05 (binomial test calculated on allele count and depth of each sample) were excluded; common dbSNP version 147 and a panel of normal (40 samples) from previous sequencing were used to annotate and filter germline variants and sequencing artifacts. The variant calls of 45 cell lines at time point 90 and the 18 cell lines explanted from mice were performed using the allele comparison strategy between the same cell line at time 0 and time point 90 and xenograft respectively. Only variants present at time point 90 (or in xenograft) were kept. Artifact removal was employed as described above. To calculate the tumor mutational burden (number of variants/Mb), only coding variants were considered. Those variants were used to predict neoantigens using previously published methods [9, 14]. Briefly, RNAseq data were used as input of “OptitypePipeline” [15] to assess the HLA status of each sample at time point 0, then NetMHC 4.0 software [16] was employed to analyze mutated peptides derived from variant calls using kmer of 8–11 length. Next, for each SNV, we modified the corresponding cDNA in the selected position and we examined the 5′ and 3′ context. The latter was set taking into account the length (in terms of amino acids) with which the putative antigen could bind HLA. We translated the cDNA and feed mutant peptide to NetMHC with the proper HLA(s). For frameshifts, we applied the same approach considering every possible peptide generated by the new frame. Finally, RNAseq data were used to annotate and then filter according to expression values (fragments per kilobase million (FPKM) > 10). Only predicted neoantigens with a strong binding affinity (Rank < 0.5) were considered for further analysis.

### Mutational analysis of patient-derived xenograft

WES of patient-derived xenografts was performed at IntegraGen SA (Evry, France). Sequenced samples included a microsatellite stable (MSS), a microsatellite unstable (MSI), and a *POLE* mutant case (5, 7, and 6 respectively). Samples were analyzed with the same bioinformatic pipeline applied to cell lines, and murine reads were first removed using Xenome [10]*.* A median depth of 130x and with 98% of the targeted region covered by at least one read was observed. All 18 PDX samples were characterized by calling alterations against the hg38 reference annotation. For each generation, with the exception of the first one, the mutational evolution was inferred by subtracting the mutations of the previous generation. Second-generation samples were compared to the first-generation samples, samples from the third generation were compared to the 2nd generation samples, and so on.

### Ploidy estimation

Gene copy-number (GCN) was calculated in a two-step approach: initially, we treated cell lines as diploid and considered the median read depth of all coding regions as the level for 2N ploidy. We also calculated the median read depth for every gene. The ratio between the two median values was then considered as the relative GCN. In the second step, to estimate the overall ploidy, we segmented all chromosomes using a custom script that implements circular binary segmentation. Finally, we exploited the distribution of allelic frequencies for individual segments to assess the absolute GCN. This was necessary since distinct ploidy levels have different expected distributions. For example, a 2N ploidy status has a bell-shaped curve with a peak of 50% and a 3N ploidy is expected to have two peaks on 33% and 66%.

### Mutational signature

Mutational signatures were calculated using the web application “Mutational Signatures in Cancer” (MuSiCa) [17]. The profile of each signature is calculated using the six substitution subtypes: C>A, C>G, C>T, T>A, T>C, and T>G (all substitutions are referred to by the pyrimidine of the mutated Watson–Crick base pair). Information on nucleotides 5′ and 3′ to each mutated base are incorporated to generate 96 possible mutation types. For each sample, a tab-separated value file was created with chromosome, position, reference, and alternate alleles. Only samples with at least 10 mutations were included. The output file of MuSiCa that includes the contribution values of 30 signatures [18] was used to create a clustermap with *seaborn,* a *Python* data visualization library, setting Euclidean metric and the average linkage method.

### Doubling time

Cell lines were passaged in vitro for a minimum of 85 to a maximum of 103 days. Each passage was performed before full confluency was achieved, and the total number of doublings was annotated for each cell model. Two parameters, number of passages (*n*) and days of culture (*t*), were used to estimate the growth rate (GR) and the doubling time (DT) assuming that every division is an independent random event; probability distribution of division is equal for all cells and it is an exponential distribution; and the number of cells in each plate before confluence is fixed (*K*). The growth rate is defined as GR = log_*n*_ (2) ÷ DT [19]. The estimated number of cells at time *t* is defined as *N*(*t*) = *N*(0) × *e*^(GR × *t*)^ where *N*(0) is the number of cells at time 0. Therefore, GR = log_*n*_(*N*(*t*) ÷ *N*(0)) ÷ *t* where *N*(*t*) ÷ *N*(0) = (*K* × 2^*n*^) ÷ (*K* × 2^0^) = 2^*n*^ and so GR = log_*n*_(2^*n*^) ÷ *t*. Finally, DT = *t* × log_*n*_ (2) ÷ log_*n*_(2^*n*^).

### RNA extraction and RNAseq analysis

Total RNA was extracted from a pellet of CRC cells (2 × 10^6^ cells) using Maxwell® RSC miRNA Tissue Kit (AS1460, Promega), according to the manufacturer’s protocol. The quantification of RNA was performed by Thermo Scientific Nanodrop 1000 (Agilent) and Qubit 3.0 Fluorometer (Life Technologies). RNA integrity was evaluated with the Agilent 2100 Bioanalyzer using the Agilent RNA 6000 Nano Kit. Total RNA (800 ng) with RNA integrity number (RIN) score between 9 and 10 was used as input to the Illumina TruSeq RNA Sample Prep Kit v2-Set B (48Rxn), according to the manufacturer’s protocol. The standard RNA fragmentation profile was used (94 °C for 8 min for the TruSeq RNA Sample Prep Kit). PCR-amplified RNA-seq library quality was assessed using the Agilent DNA 1000 kit on the Agilent 2100 BioAnalyzer and quantified using Qubit 3.0 Fluorometer (Life Technologies). Libraries were diluted to 10 nM using Tris-HCl (10 mM pH 8.5) and then pooled together. Diluted pools were denatured according to the standard Illumina protocol, and 1.8 pM were run on NextSeq500 using high output Reagent cartridge V2 for 150 cycles. A single-read 150-cycle run was performed. FastQ files produced by Illumina NextSeq500 were aligned using MapSplice2 [20] transcriptome-aware aligner using hg38 assembly as reference genome. The resulting BAM files were post-processed to translate genomic coordinates to transcriptomic ones and to filter out alignments carrying insertions or deletions (which RSEM does not support) or falling outside the transcriptome regions. The post-processed BAM alignment was given as input to RSEM [21] for gene expression quantification using GENCODE v22 as gene annotation.

### Differential expression analysis

The abundance quantification generated with RSEM provides the FPKM and the expected counts for each gene. The latter was used to perform genes differential expression analysis with DESeq2 R package (library Bioconductor) [22] given two distinct groups of interest, one of which considered as the reference. Genes were considered as differentially expressed if the adjusted *p* value was less than 0.05, and the log2 fold change was less or equal to −1 (if median FPKM value of the reference group was greater or equal to 10), or the log2 fold change was greater or equal to 1 (if median FPKM of the target group was greater or equal to 10). The analyses were performed between the following groups: MSI vs MSS (reference), hypermutated vs non-hypermutated (reference), and “EVOLVING-CRC” vs “STABLE-CRC” (reference). The hypermutated group included MSI and MSS POLE-mutated cell lines (18 samples). EVOLVING-CRC group included all samples with at least 10 alterations acquired per day. A multi-factor configuration of the expression analysis was designed including extra variables of interest such as growth rates or the number of mutations normalized to doubling time.

### Pathway analysis

Genes differentially expressed were then analyzed with g:Profiler [23], an online pathway analysis tool that takes a list of genes and assigns them to different families of biological functions. We set the query options to select significant biological processes only, and we retained (for further analysis) only the topmost families of the hierarchy (depth 1).

### Xenograft mouse model

Each CRC cell line (5 × 10^6^ cells) was injected subcutaneously into both flanks of two 6-week-old female NOD (nonobese diabetic)/SCID (severe combined immunodeficient) mice (Charles River Laboratory). Tumor size was measured twice a week and calculated using the formula: *V* = ((*d*)^2^ × (*D*)) ÷ 2 (*d* = minor tumor axis; *D* = major tumor axis). Tumors were explanted when they reached a volume of 1000 mm^3^. The investigators were not blinded, and measurements were acquired before the identification of the cages.

### Patient-derived mouse model

Tissue from hepatic metastasectomy of CRC patients was collected at surgery and implanted in NOD-SCID mice as described previously [24]. When reaching a volume of 1500–2000 mm^3^, the tumors were explanted, fragmented, and serially passaged in new mice. At each passage, part of the material was frozen for molecular analyses. Samples’ genetic identity was determined by Sequenom-based analysis of 24 highly variable SNPs of germline DNA (Table 2), confirmed by analyzing pre-implantation tumor material, and then validated every second passage in mice. The study population consisted of matched tumor and normal samples from 3 CRC patients that underwent surgical resection of liver metastases at the Candiolo Cancer Institute (Candiolo, Torino, Italy) and at the Mauriziano Umberto I Hospital (Torino) between 2009 and 2013. Patients signed informed consent, and the study was approved by the relevant institutional Ethics Committees.

**Table 2:** List of SNPs used to identify patient-derived xenografts

Probe	SNP	GENE	Chr	Functional consequence
AMG_mid100				
rs11017876	[A\G]	DOCK1	10:127402700	Intron variant
rs1106334	[C\T]		8:70100576	
rs1155741	[C\T]	ITGA9	3:37585621	Intron variant
rs11655512	[A\G]	LOC339260	17:20948422	Intron variant
rs11940551	[G\T]		4:27160856	
rs1210110	[A\G]	PRDM2	1:13770326	Intron variant
rs1364054	[C\T]	LINC00299	2:8038605	Intron variant
rs1528601	[C\G]		16:51064516	
rs161792	[A\T]	LOC101928166	3:152181915	Intron variant
rs17272796	[C\T]	PLCL2	3:17035776	Intron variant
rs242076	[C\T]	SYN3 - TIMP3	22:32833844	Intron variant
rs4775699	[C\T]	SEMA6D	15:47581352	Intron variant
rs4793172	[A\T]	DCAKD	17:45054112	Intron variant
rs4905366	[A\G]		14:95636762	
rs6603251	[C\T]	PPP2R3B	Y:359845	Intron variant
rs6734275	[A\G]	LOC105374785	2:67014042	Intron variant
rs685449	[A\T]	RGS17	6:153023396	Intron variant
rs7555566	[A\G]	KAZN	1:14478378	Intron variant
rs7584993	[A\C]		2:222981224	
rs7808249	[A\G]	CROT	7:87354399	Intron variant
rs9293511	[C\T]	LOC105379072	5:89120537	Intron variant
rs9352613	[A\G]		6:78714716	
rs9572094	[C\T]	LOC105370159	13:34678745	Intron variant

### Western blotting analysis

Proteins were extracted by solubilizing the cells in boiling SDS buffer (50 mM Tris-HCl [pH 7.5], 150 mM NaCl, and 1% SDS). Samples were boiled for 5 min at 95 °C and sonicated for 10 s. Extracts were clarified by centrifugation, normalized with the BCA Protein Assay Reagent kit (Thermo). Equal amounts of proteins (20 μg) were loaded in each lane. Proteins were separated by PAGE and transferred to nitrocellulose sheets. Western blot detection was performed with enhanced chemiluminescence system (GE Healthcare) and peroxidase-conjugated secondary antibodies (Amersham). The following primary antibodies were used for western blotting: anti-beta2 Microglobulin [EP2978Y] (ab75853, Abcam), anti-MLH1 (ab92312, Abcam), anti-MSH2 (ab70270, Abcam), anti-MSH6 [EPR3945] (ab92471, Abcam), anti-MSH3 PA527864, Invitrogen, anti-PMS2 EPR3947 (Cell Marque Corporation, USA), anti-actin (I-19) (sc1616, Santa Cruz), and anti-HSP 90α/β (H-114, sc-7947, Santa Cruz). Images were acquired with Chemidoc (Biorad), and western blot band intensity was analyzed using Image Lab software (Biorad).

## Results

**Fig. 1:**
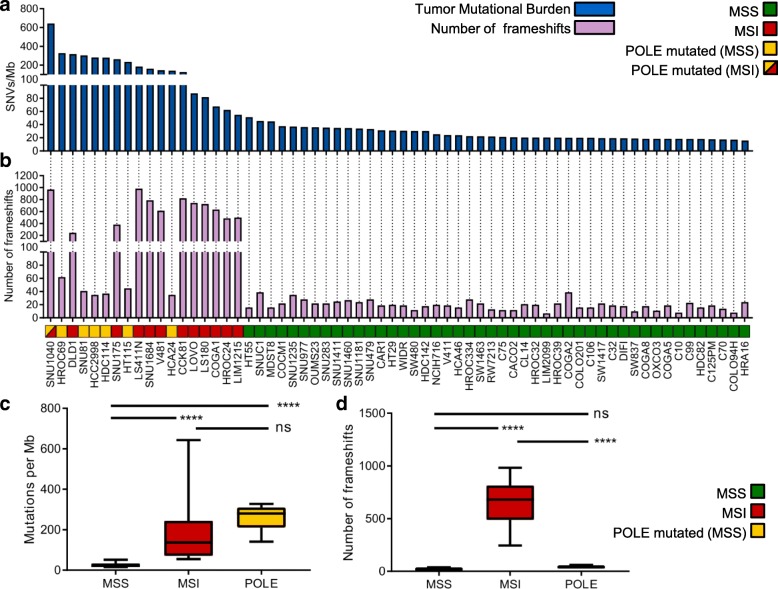
Analysis of mutational burden in a panel of 64 CRC cell lines. Mutational characterization and comparison of SNVs and frameshifts among MSS (46 samples), MSI (12 samples), and POLE mutated (6 samples) of CRC models. **a** The distribution of SNVs per Mb of coding DNA at time 0 is shown for each cell line. **b** The number of frameshift mutations at time 0 is shown for each cell line. **c** The number of SNVs per each group is shown (“MSS” refers to MSS cells without POLE mutations; “MSI” includes MSI cells, as well as the SNU1040 cell line which is both MSI and POLE mutated; “POLE” includes only MSS cell lines carrying a POLE mutation). **d** The number of frameshifts per group is shown. The center line of each box plot indicates the median. *p* < 0.0001

We selected from our database 64 CRC cell lines designed to recapitulate clinically relevant characteristics of CRC patients (Table 1 and Additional file 1: Figure S1a). Whole exome sequencing and RNAseq were performed on all models. Using previously developed computational tools and bioinformatic algorithms [13, 14, 25, 26], we measured mutational burden (alterations per Mb) assessing both SNVs and frameshifts (Fig. 1a, b, Additional file 2). Scrutiny of genomic alterations highlighted that MSI cell lines and those carrying known *POLE* hotspot mutations had higher number of mutations per Mb as compared to MSS cell lines (Fig. 1a). The type of DNA repair alterations occurring in each model affected the nature of mutations: MSI cells displayed a higher number of frameshifts and indels than *POLE* mutant cell lines; the opposite was true for SNVs (Fig. 1c, d).

Alterations in MMR and *POLE* genes are listed in Table 3 and Additional file 1: Figure S1b. The cell line with the highest number of variants (SNU1040) carried inactivating alterations in both *MLH1* and *POLE* (Additional file 1: Figure S1b). Altogether, these results are consistent with what has been reported in CRC patients carrying alterations in the MMR DNA repair pathway, indicating that the cell models included in this study broadly recapitulate what is observed in clinical specimens [27].

**Table 3:**
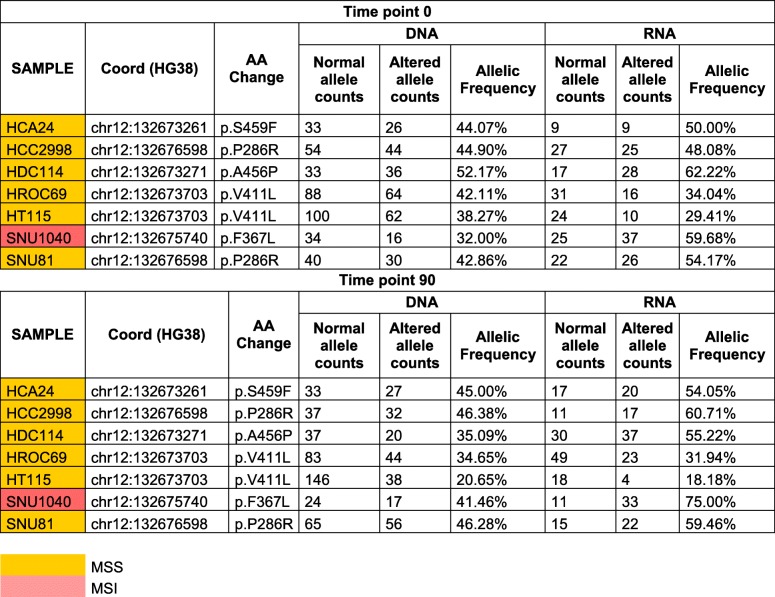
POLE mutations in CRC cells

**Fig. 2:**
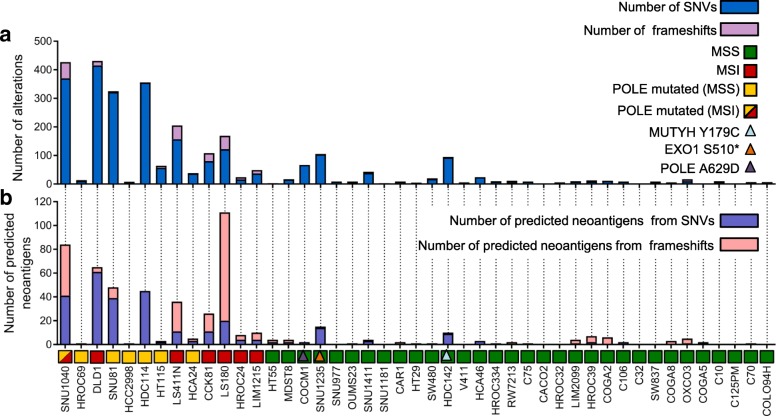
In vitro evolution of mutational landscape in 45 CRC cell lines. Mutational characterization of CRC cells after 90 days of culture (T90*)* in vitro. **a** Bar charts show the number of novel alterations (SNVs and frameshifts) acquired at T90 (not present at T0) for each cell line. **b** The number of predicted neoantigens (see the “Methods” section) is shown. Each bar represents putative neoepitopes derived from SNVs and frameshifts

To assess whether, and to what extent the basal mutational profiles (Time 0: T0) evolved over time, we passaged 45 cell lines for 90 days and collected a second set of samples (Time 90: T90) (Additional file 1: Figure S2). These were subjected to WES and analyzed using the computational pipeline described above. Across all cell lines globally, the total mutational burden was similar between T0 and T90 (Additional file 1: Figure S3). However, when the T0 and T90 mutational profiles were compared, prominent differences were detected among models sharing specific DNA repair defects (Fig. 2a). Specifically, the mutational landscapes of most MSI and *POLE* mutant cells evolved very rapidly through the generation of novel SNVs and frameshifts (Fig. 2a). On the contrary, the majority of MSS models showed more stable profiles (Fig. 2a). We sought to minimize confounding effects due to differences in cell-intrinsic doubling times (Table 1); we therefore calculated the doubling time of all cell models (Table 1, Additional file 1: Figure S4). Notably, evolvability trends remained apparent after normalization for doubling time (Additional file 1: Figure S5). We designated rapidly evolving CRC cells as EVOLVING-CRC and evolutionary stable CRC cell as STABLE-CRC (Table 1).

We empirically define EVOLVING-CRCs as those cells that acquire 10 alterations (or more) per day after normalizing mutation data to the doubling time of cell lines (Table 1). Moreover, EVOLVING-CRCs often carried alterations in multiple genes involved in distinct DNA repair functions, suggesting that defects in several DNA damage response pathways might be co-selected (Additional file 1: Figure S1b). The expression of MMR genes was assessed by western blot at T0 and T90, and no differences were observed (Additional file 1: Figure S6).

The genome of four CRC lines classified as MSS (SNU1235, COCM1, HDC142, and SNU1411) exhibited dynamic mutational profiles (Fig. 2). In an attempt to decipher the molecular basis of these findings, whole exome data of the outliers were carefully examined, focusing on genes previously implicated in DNA repair pathways that are not routinely subjected to scrutiny in CRC patients. We found that SNU1235 and HDC142 models carried biallelic alterations in the *EXO1* (S510*) and *MUTYH* (S179C) genes, respectively. The exonuclease EXO1 is implicated in both MMR (it binds MLH1) and base excision repair [28], while *MUTYH* encodes a DNA glycosylase that is involved in oxidative DNA damage repair and is part of the base excision repair pathway [29]. Germline mutations in *MUTYH* cause MUTYH-associated polyposis (MAP) [30]. Scrutiny of the COCM1 exome revealed a *POLE* variant (A629D). A629 is localized in a region of *POLE* highly conserved during evolution (Additional file 1: Figure S7). The A629D change is potentially damaging according to the SIFT [31] and Polyphen [32] algorithms, which predict the putative impact of amino acid substitutions on human proteins using structural and comparative evolutionary considerations.

We next addressed how longitudinal evolution of CRC cell genomes affected their predicted neoantigen profile. To this end, WES, RNAseq, and HLA prediction data were combined as previously described [9]. In detail, we identified genomic variants that satisfied three criteria: (i) emerged over time, (ii) occurred in transcribed genes, and (iii) scored positively when HLA I matching algorithms were applied. The variants that emerged after deploying the above computational pipeline were classified as putative neoantigens (Fig. 2b). Hypermutated and EVOLVING-CRC cells displayed higher levels of putative neoantigens compared to slowly evolving CRC cells (Fig. 2b). Moreover, and consistent with their predicted effects on antigenicity, a high prevalence of indels and associated frameshifts, which occur in MSI CRCs, translated into higher numbers of predicted neoantigens in this subset (Fig. 2b).

**Fig. 3:**
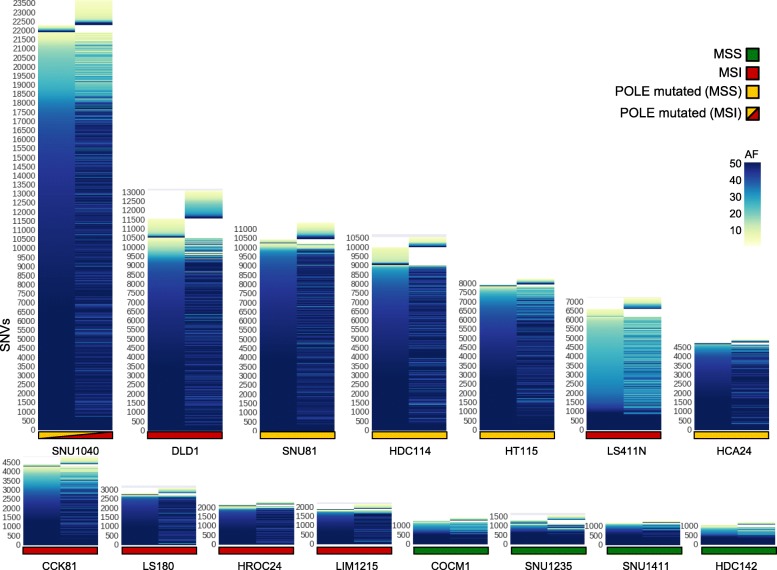
Lost and gained mutations across evolving CRC cell lines. For each CRC model, the allelic frequency of SNVs at T0 and T90 are shown. Mutations were called against the reference genome (hg38) with allelic frequency > 1. The *y*-axis reports all the mutations found in each cell line, whereas the time points data are reported on *x*-axis

Next, we studied whether in parallel to mutation gains we could also detect loss of variants over time. For this reason, we tracked lost and gained alteration in “evolving” cell lines over time. As expected, variants that did not change over time showed high allelic frequency, likely reflecting their clonal (trunk) status. Mutations that emerged or were lost showed lower allelic frequency (Fig. 3).

Mutational signatures are characteristic combinations of mutation types arising from mutagenesis processes such as alterations in DNA replication, exposure to DNA damaging agents, tissue culture conditions, and DNA enzymatic editing [18]. In human tumors, over 30 mutational signatures have been identified, a subset of which are linked to defective DNA repair pathways. For example, signatures 6, 15, 20, and 26 are associated with MMR defects and signature 10 is linked to inactivating mutation in the proofreading domain of DNA polymerases, while signature 18 appears to explain the rise of 8-oxoG:A mismatches due to *MUTYH* biallelic alteration [33].

We reasoned that the remarkable evolvability observed in a subset of CRC cells might be reflected in their mutational signatures. To test this, we first identified mutational signatures at T0. As expected, MSI cells displayed signatures 6, 15, 20, and 26, while *POLE* mutant cells showed primarily mutational signature 10 (Additional file 1: Figure S8).

**Fig. 4:**
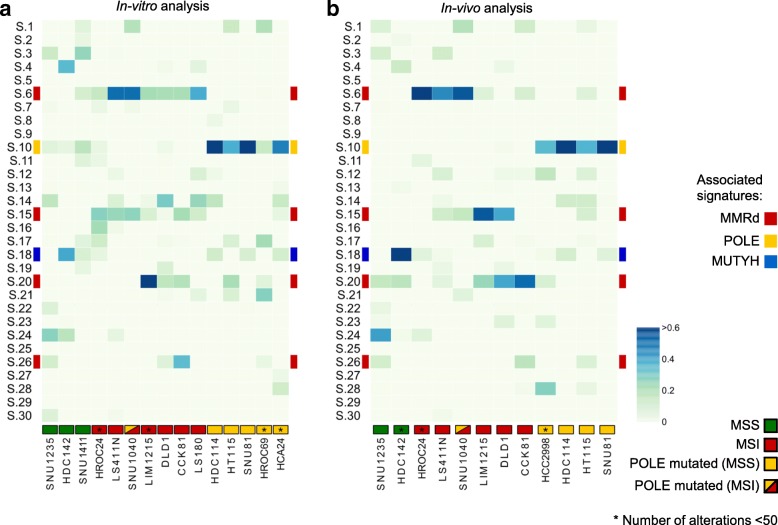
Mutational signatures associated with alterations emerging during in vitro or in vivo CRC propagation. Analysis of 30 validated cancer-associated mutational signatures in hypermutated/rapidly evolving CRC cell lines. Signatures associated to MMR-deficient (6, 15, 20, 26), *POLE*-dependent (10), and *MUTYH*-associated polyposis (18) are highlighted. Analysis and clustering were performed as reported in the “Methods” section. **a** Heatmap of signature contributions during replication of CRC cells in vitro by analyzing alterations acquired at T90. **b** Heatmap of signature contributions during replication of the CRC cells in vivo by comparing xenograft tumors to the corresponding cells at T0 (see the “Methods” section for detailed information)

We next assessed which signatures were acquired (remained active) during replication of the cells in vitro by comparing samples collected at T0 and T90. We found that in most instances, DNA alterations linked to MMR and *POLE* defects continued to occur over time, indicating that the corresponding DNA repair capabilities were permanently disabled (Fig. 4a).

Replication of cancer cell populations in 2D is thought to encounter little or no selective pressure as the cells are cultured in the same conditions for many generations before the experiment is started. To monitor mutational and neoantigen evolution under more stressful (selective) conditions, CRC cells including MSS, MSI, and *POLE* models were transplanted in immunodeficient (NOD SCID) mice and allowed to grow until they reached approximately 1000 mm^3^ in size, after which tumors were excised. Although NOD SCID mice have no adaptive immunity, the mouse stromal microenvironment and elements of cellular innate immunity are known to affect the growth of human cancer cells in vivo [34]. DNA samples were obtained before implantation and at the end of the experiment. WES was performed, and the data were analyzed with the same bioinformatic pipeline applied to cells grown in vitro. The mutational profiles revealed higher evolutionary rates in vivo than in vitro (Additional file 1: Figure S9a, b). This translated into increased levels of predicted neoantigens in vivo (Additional file 1: Figure S9c). Notably, mutational signatures linked to MSI status and *POLE* mutations were more marked in vivo than in vitro (Fig. 4b, Additional file 1: Figure S10). We then assessed whether the mouse microenvironment exerts selection on the cells expanded in vivo and compared the results to cells passaged in vitro. To this end, we characterized the ratio between non-synonymous and synonymous mutations in vitro and in vivo. We detected very limited or no selection in cells passaged in vitro (ratio 3:1). Instead, in vivo the ratios for lost and gained mutations were 1:1 and 2:1, respectively, indicating a purifying selection (Additional file 1: Figure S11). These findings suggest that when cells are transplanted in mice they are subjected to environmental selection.

**Fig. 5:**
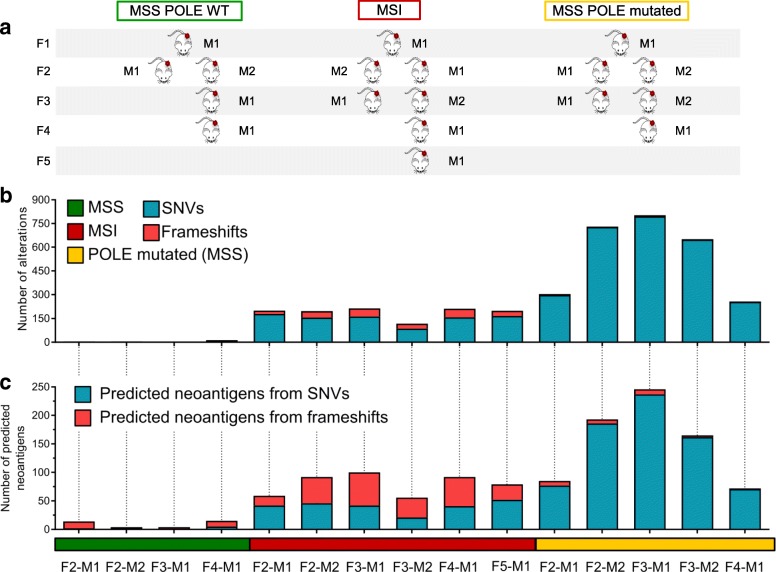
Genomic evolution in patient-derived xenografts. Phylogeny of the indicated patient-derived xenograft and their molecular characterization. **a** MSS, MSI, and *POLE* mutant samples were serially transplanted for at least four generations (F1–F4) in NOD/SCID mice as shown. Samples collected at each passage were subjected to WES. **b** WES data of each generation were compared with those obtained from the previous generation. Bar graphs show de novo acquired SNVs and frameshifts at each generation. **c** The number of predicted neoantigens in each PDX is shown. Each bar represents putative neoepitopes derived from SNVs and frameshifts (see the “Methods” section for detailed information)

**Fig. 6:**
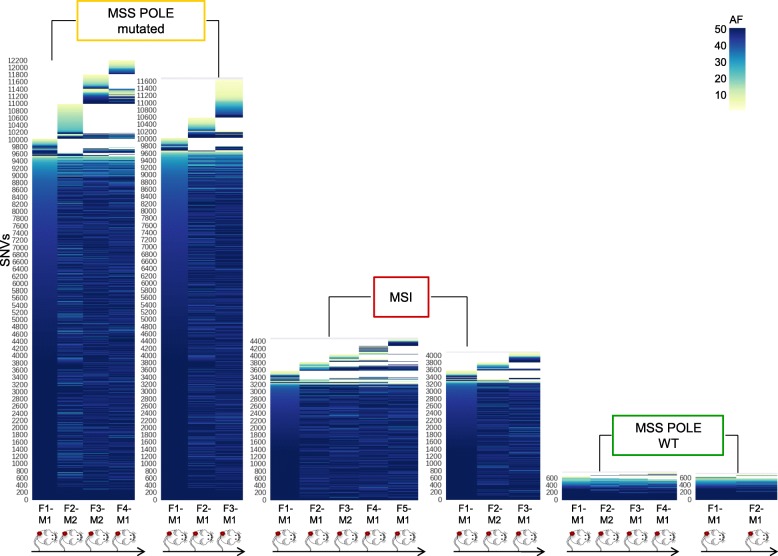
Lost and gained mutations across the indicated PDX generations. The color code defines allelic frequencies of acquired SNVs at each generation (with allelic frequency > 1). The *y*-axis lists all SNVs identified in each branch; the mouse generation (genealogy) is reported on the *x*-axis

Next, we asked whether the evolutionary trajectories observed in CRC cells with alterations in DNA repair pathways also occurred in human CRC with analogous molecular profiles. To this end, we selected MMR-proficient, MMR-deficient, and POLE mutant cases (Table 4) from our extensive patient-derived CRC xenograft biobank [35]. Each model was serially transplanted for at least four generations in immunodeficient mice as described in the phylogenetic tree (Fig. 5a). Samples collected at each transplantation were subjected to WES. In some instances, simultaneous transplantation of the same tumor in two animals allowed acquisition of independent measurements for each generation. NGS data were analyzed with the bioinformatic pipeline applied to cells grown in vitro. These experiments revealed remarkable differences in the evolvability of MSS, MSI, and *POLE* CRC models in vivo and indicated that these characteristics also occurred in patient-derived CRC samples (Fig. 5b, c). As expected, high-frequency (clonal-trunk) variants were conserved across generations. Interestingly, the in vivo results differ from those obtained in cell models in vitro. We find that in PDX models, not only sub-clonal but also clonal populations can emerge in the subsequent generation of colorectal cancers with DNA repair defects (Fig. 6).

**Table 4:** Molecular characterization of patient-derived xenografts

Sample	MSI status	BRAF	KRAS	NRAS	MLH1	MSH2	MSH3	MSH6	PMS2	POLE
CRC106	MSI	V600E	WT	WT	WT	WT	WT	R577H / p.T1085Tfs7*	WT	WT
CRC542	MSS	WT	WT	WT	WT	WT	WT	WT	WT	WT
CRC371	MSS	WT	WT	E132K	R265C	WT	WT	E946*	WT	V411 L

*Only non-synonymous variants present in COSMIC database are reported

Furthermore, in MSI and *POLE* patient-derived xenografts, the mutational signatures were continuously (re)generated and could be clearly recognized (Additional file 1: Figures S12 and S13). In non-mutator (slow evolving) cell lines, very few mutations emerged over time, and thus, the possibility to assess mutational signatures was limited. Because of this, in the slowly evolving models, we were unable to reliably generate mutational signatures.

**Fig. 7:**
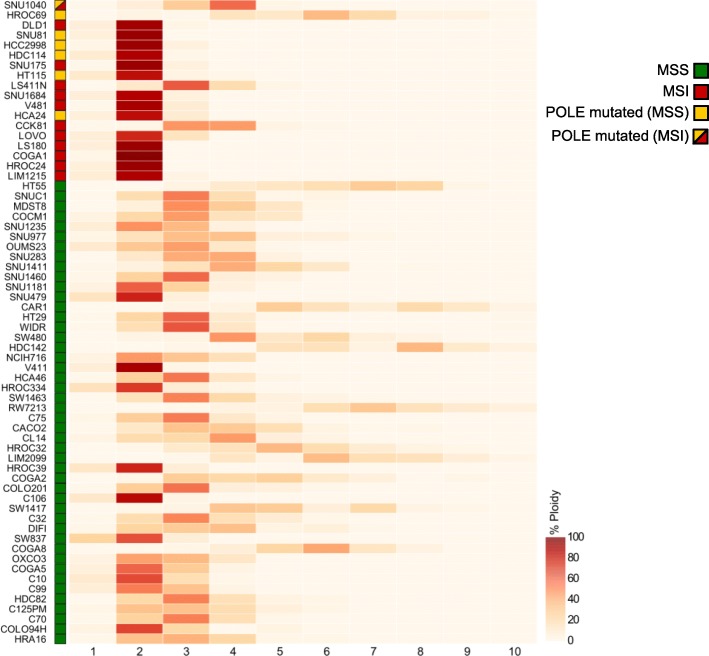
Analysis of cell ploidy in a panel of 64 CRC cell lines. Heatmap showing distribution of ploidy for every segmented region in each cell line. Samples are sorted from most to less mutated as reported in Fig. 1. The percentage (ploidy) is calculated as described in detail in the “Methods” section

Distinct subsets of CRCs can be recognized based on histological characteristics, as well as their genomic, epigenetic, and transcriptional profiles. As a result, CRC can be classified into specific subsets, which are often correlated with divergent clinical outcomes [36, 37]. The rate of genomic evolution and the dynamics of neoantigen profile have not yet been systematically explored as a method to classify CRC. We therefore asked whether any molecular traits (beyond alterations in DNA repair genes) could distinguish EVOLVING-CRC and STABLE-CRC. To address this question, we performed unbiased gene copy number and transcriptional comparative analyses of CRC cell lines. As previously reported, MSI CRC cells typically carried a close to diploid chromosomal status, while MSS showed elevated aneuploidy (Fig. 7) [38]. Interestingly, the most rapidly evolving *POLE* mutant lines, SNU81 and HDC114, also displayed a diploid prevalent phenotype. Nonetheless, copy number and ploidy status could not distinguish “EVOLVING” and “STABLE” CRC models.

**Fig. 8:**
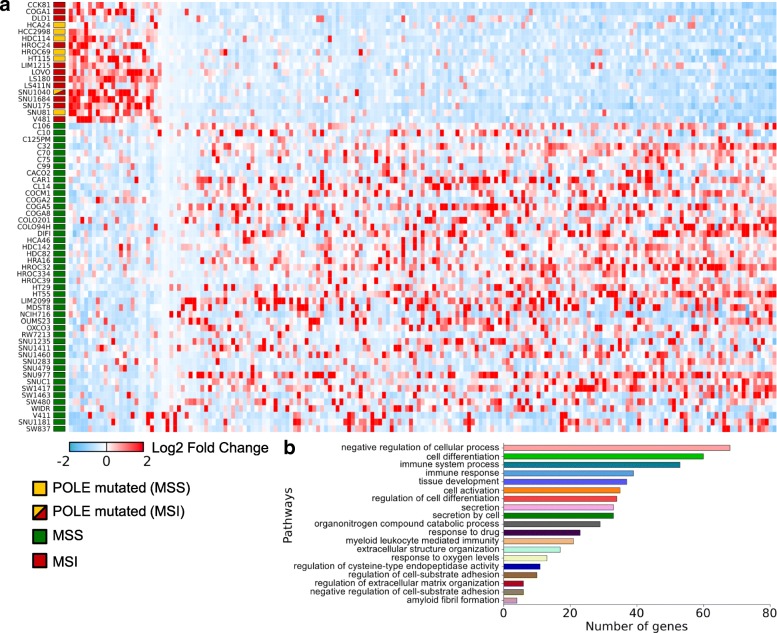
Transcriptional analysis of CRC cell lines. Differential expression analysis between hypermutated and non-hypermutated cells. **a** 183 unique genes differentially expressed between hypermutated (MSI/*POLE*) versus non-hypermutated CRC cells (MSS). Log2 expression values along with the mean change in expression are shown. **b** Pathway analysis of genes differentially expressed between hypermutated versus non-hypermutated CRC cells using g:Profiler application (see the “Methods” section)

**Fig. 9:**
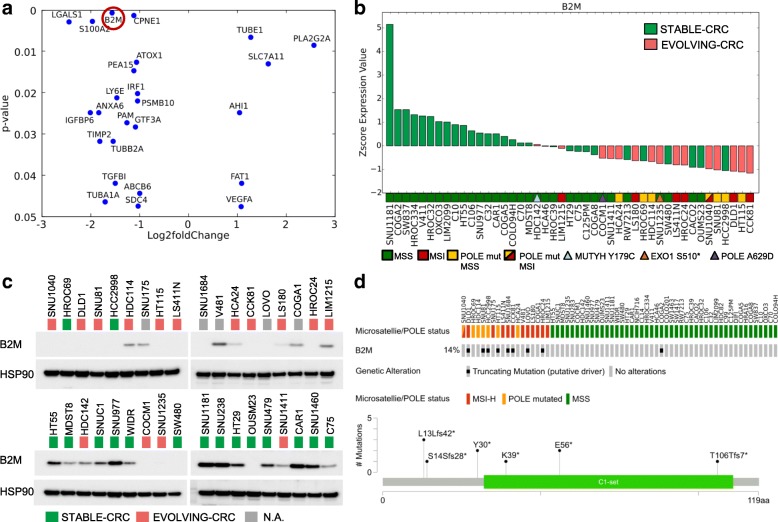
Beta2 microglobulin (B2M) expression is downregulated in EVOLVING-CRC. Transcriptional and protein levels of the B2M gene. **a** Genes differentially expressed in EVOLVING-CRC relative to STABLE-CRC with a significant *p* value (*p* < 0.05). **b** Waterfall chart showing B2M expression at RNA level across a panel of 45 CRC cell lines. **c** Western blot analysis of B2M expression. In gray are highlighted samples for which T90 sequencing were not available. Blots were reprobed with anti-HSP90 antibody to confirm equal loading. **d** *B2M* gene alterations on 64 CRC cell lines at T0 (upper panel) and codon affected (lower panel)

Next, we performed RNAseq on the entire dataset to explore whether transcriptional profiles could classify rapidly evolving CRC lines. Differential analysis of RNAseq data was initially performed comparing the MSS and MSI sample groups. The list of differentially expressed genes was consistent to results previously reported in this setting, and 168 genes were differentially expressed between these two groups (Table 5) [39]. Next, we evaluated genes differentially expressed in hypermutated versus non-hypermutated cells, grouping together MSI- and *POLE*-mutated cell lines and comparing them to the MSS lines (Fig. 8a). Notably, proteins associated with immune response and predominantly with antigen-presenting and antigen recognition functions were consistently downregulated in cell lines with high mutational burden (Fig. 8b). Next, we compared EVOLVING and STABLE CRC models. The number of genes differentially expressed with significant *p* value was smaller due to the reduced number of available samples (Fig. 9a). Beta-2 microglobulin (B2M) was downregulated in most EVOLVING as compared to STABLE CRCs (Fig. 9b, c). Downregulation of B2M was confirmed at the protein level (Fig. 9c) and was frequently associated with premature stop codons in the *B2M* gene (Fig. 9d). Interestingly, the four MSS models (COCM1, SNU1235, SNU1411, and HDC142) with low mutational burden but dynamic mutational profile also displayed low levels of B2M (Fig.9b, c). Comparison of EVOLVING and STABLE CRC models pinpointed other genes differentially expressed including CPNE1, IRF1, and PMSB10. These genes are also involved in immune-related processes and their downregulation might similarly reduce immune surveillance of EVOLVING CRCs (Fig. 9a and Additional file 1: Figure S14). We next performed the analysis showed in Fig. 9a in a multivariate fashion taking into account the growth rates of the cells or the number of mutations normalized to the doubling time. The number of statistically significant genes in the multivariate analyses (Additional file 1: Figure S15) was lower but consistent with the findings of Fig. 9a. In the future, it would be interesting to assess whether the differential expression of genes in fast evolving CRC models has a functional impact. This aspect cannot be causally predicted at this stage.

**Table 5:** List of genes differentially expressed in the indicated cell lines

gene_id	Log2 fold change	*p* adj.
MSS vs MSI		
ABCB6	− 1.12	0.01
ACAD11	1.18	0.00
ACOT8	− 1.04	0.00
AHCYL2	1.12	0.02
ALDH6A1	1.34	0.00
AMACR	− 1.54	0.01
AOC1	− 1.57	0.04
APBB3	1.04	0.00
ARHGAP18	− 1.01	0.02
ATOX1	− 1.00	0.01
ATP5E	− 1.22	0.00
ATP9A	− 1.77	0.00
B2M	− 1.58	0.00
B4GALT5	− 1.14	0.00
BCL2L15	− 1.73	0.01
BCL9L	− 1.09	0.02
BPTFP1	1.09	0.03
C15orf52	− 1.28	0.05
C20orf24	− 1.14	0.00
C6orf48	1.05	0.01
CAPG	− 1.30	0.02
CCDC71L	− 1.08	0.02
CCND3	− 1.26	0.00
CCPG1	1.16	0.02
CD59	− 1.01	0.03
CD82	− 1.37	0.03
CDKN2A	− 3.20	0.00
CEACAM1	− 1.89	0.00
CEACAM6	− 1.71	0.05
CGN	− 1.06	0.02
CHKA	− 1.13	0.00
CHMP4B	− 1.16	0.00
CLDN1	− 1.37	0.05
CLDN3	− 1.58	0.01
COL17A1	− 1.61	0.03
COTL1	− 1.61	0.00
CPNE1	− 1.05	0.00
CPT1B	1.13	0.00
CTSA	− 1.10	0.00
CTSD	− 1.02	0.03
CTSH	− 1.22	0.03
CTSS	− 2.46	0.00
CTSV	− 1.81	0.00
CTSZ	− 1.08	0.01
CUEDC1	− 1.33	0.02
CXXC5	− 1.24	0.01
CYB5D1	1.24	0.00
CYFIP2	1.37	0.03
DAB2IP	− 1.14	0.00
DCBLD2	− 2.43	0.00
DDX27	− 1.02	0.00
DNTTIP1	− 1.25	0.00
DPEP1	− 1.88	0.04
DYNLRB1	− 1.04	0.00
EDN1	− 1.79	0.01
EMP1	− 1.47	0.03
EPB41L1	− 1.09	0.01
EPB41L4A-AS1	1.04	0.01
EPS8L1	− 1.13	0.03
FCGRT	− 1.69	0.00
FECH	1.07	0.00
FKBP1A	− 1.02	0.01
FZD7	− 1.02	0.03
GABBR1	1.70	0.01
GABRE	− 1.83	0.00
GCAT	1.39	0.00
GLS2	1.38	0.00
GNE	− 1.13	0.04
GSN	− 1.30	0.03
HELZ2	− 1.14	0.01
HIST1H2AC	− 1.36	0.01
HIST1H2BD	− 1.92	0.00
HIST1H2BK	− 1.48	0.00
HIST2H2AA3	− 1.13	0.03
HIST2H4A	− 2.11	0.00
HSPA1A	− 1.83	0.02
HSPB1	− 1.41	0.03
HSPH1	− 1.43	0.00
IDH2	− 1.01	0.04
IDS	− 1.30	0.02
IFI6	− 1.76	0.03
IRF1	− 1.02	0.01
KRT20	− 1.73	0.04
KRT23	− 4.08	0.00
LAMC2	− 1.77	0.00
LFNG	− 1.19	0.02
LGALS1	− 2.88	0.00
LINC01089	− 1.05	0.01
LIPG	1.38	0.00
LPCAT2	− 1.11	0.01
LRRC75A-AS1	1.01	0.04
LRRC8A	− 1.23	0.00
LTBP3	− 1.37	0.01
LY6E	− 1.41	0.02
LY75	− 1.60	0.01
MACF1	− 1.23	0.01
MALL	− 1.20	0.03
MAP7D1	− 1.03	0.02
MAPRE3	− 1.11	0.02
MDM2	1.37	0.00
MGLL	− 1.35	0.01
MIR4435-2HG	− 1.09	0.05
MMP14	− 1.53	0.02
MOCOS	1.18	0.01
MORC4	1.15	0.00
MUC20	− 1.64	0.02
MYBL2	− 1.13	0.01
MYL5	1.04	0.01
NABP1	− 1.35	0.01
NDUFC2	− 1.22	0.00
OXR1	− 1.12	0.01
PDP1	− 1.01	0.03
PEA15	− 1.29	0.00
PFDN4	− 1.04	0.00
PIGT	− 1.00	0.00
PLA2G6	1.17	0.00
PLS3	− 1.21	0.03
PMEPA1	− 2.81	0.00
PML	− 1.04	0.00
POLE4	− 1.01	0.01
PPM1M	1.46	0.00
PPP1R14D	− 1.98	0.01
PPP1R18	− 1.27	0.01
PRADC1	− 1.19	0.01
PRAP1	− 1.79	0.04
QPCT	− 1.89	0.02
QPRT	− 2.16	0.00
REG4	2.81	0.00
ROMO1	− 1.14	0.01
RPL22L1	1.62	0.00
RPL32P29	1.31	0.00
S100A11	− 1.62	0.00
S100A2	− 2.35	0.00
S100A4	− 2.70	0.00
SDC4	− 1.30	0.00
SESN2	1.23	0.01
SGK2	− 1.40	0.04
SLC20A2	− 1.07	0.01
SLC2A1	− 1.22	0.04
SLC39A5	− 1.93	0.02
SLC6A6	− 1.40	0.00
SNHG8	1.01	0.01
SNORA73B	1.12	0.02
SPINK1	− 2.25	0.01
STAT1	− 1.06	0.01
SULT2B1	− 1.78	0.01
SUPT4H1	1.22	0.00
SYT7	− 1.82	0.00
TCF7	− 1.03	0.04
TFF1	2.17	0.02
TGFBI	− 2.43	0.00
TIMP2	− 2.16	0.01
TM4SF1	− 1.70	0.01
TMEM52	1.08	0.03
TMPRSS4	− 1.35	0.04
TNFSF9	2.01	0.00
TRIM7	1.24	0.05
TSPAN6	− 1.49	0.00
TUBA4A	− 1.11	0.00
TUBE1	1.12	0.01
TXNDC9	− 1.04	0.00
UCA1	− 1.79	0.03
UCP2	− 1.38	0.03
UNC13D	− 1.81	0.01
VAMP8	− 1.09	0.00
VOPP1	− 1.13	0.01
ZMYND8	− 1.11	0.00
ZNFX1	− 1.08	0.00
Hypermutated vs non-hypermutated		
ABHD12	− 1.10	0.00
ACOT8	− 1.09	0.00
AHCYL2	1.00	0.01
AKR1C3	− 1.52	0.02
ALDH6A1	1.30	0.00
AMN	1.06	0.04
ANXA6	− 1.94	0.00
AOC1	− 1.96	0.00
ARHGEF10	1.15	0.04
ARL4C	− 1.56	0.01
ATOX1	− 1.12	0.00
ATP5E	− 1.31	0.00
ATP8B1	1.09	0.00
ATP9A	− 1.77	0.00
B2M	− 1.82	0.00
BCAS4	− 1.06	0.00
BCL2L1	− 1.02	0.00
BNIP3	1.31	0.02
C15orf52	− 1.10	0.04
C20orf24	− 1.21	0.00
C2orf54	− 2.00	0.00
C7orf50	− 1.20	0.00
CAPG	− 1.02	0.03
CCDC71L	− 1.00	0.01
CD59	− 1.13	0.00
CD82	− 1.31	0.01
CDKN2A	− 1.43	0.03
CEACAM1	− 1.42	0.00
CFD	1.65	0.00
CHKA	− 1.17	0.00
CHMP4B	− 1.25	0.00
CLDN3	− 1.45	0.00
COL17A1	− 1.33	0.03
COTL1	− 1.67	0.00
CPNE1	− 1.09	0.00
CST3	− 1.29	0.00
CTSA	− 1.01	0.00
CTSD	− 1.27	0.00
CTSH	− 1.31	0.00
CTSS	− 2.26	0.00
CTSV	− 2.11	0.00
CTSZ	− 1.05	0.00
CUEDC1	− 1.59	0.00
CXXC5	− 1.01	0.01
CYB5D1	1.17	0.00
CYTOR	− 1.42	0.00
DBNDD2	− 1.06	0.00
DCBLD2	− 2.20	0.00
DGAT2	− 1.04	0.01
DHRS3	− 1.09	0.01
DNTTIP1	− 1.23	0.00
DYNLRB1	− 1.25	0.00
EDN1	− 1.27	0.03
EHD1	− 1.01	0.00
EMP1	− 1.60	0.00
EPB41L1	− 1.06	0.00
EPS8L1	− 1.20	0.00
FAH	− 1.28	0.00
FAM84B	− 1.10	0.02
FCGRT	− 1.60	0.00
FECH	1.27	0.00
FKBP10	− 1.41	0.04
FKBP1A	− 1.16	0.00
FUNDC2	− 1.04	0.00
FUNDC2P1	− 1.08	0.00
FZD7	− 1.19	0.00
GABARAPL1	− 1.35	0.01
GABRE	− 1.53	0.00
GCAT	1.01	0.01
GNE	− 1.03	0.02
GPC1	− 1.01	0.05
GSN	− 1.75	0.00
HELZ2	− 1.02	0.01
HIST1H2BD	− 1.04	0.01
HIST2H4A	− 1.38	0.00
HSD11B2	− 1.11	0.03
HSPB1	− 1.20	0.02
IDS	− 1.60	0.00
IFI27	− 1.48	0.03
IFI27L2	− 1.03	0.03
IFI6	− 1.86	0.00
IGFBP4	− 1.15	0.02
IL33	1.58	0.04
IRF1	− 1.28	0.00
ISG15	− 1.13	0.04
ITGA3	− 1.23	0.01
ITGB5	− 1.09	0.00
KIFC3	− 1.20	0.04
KLK6	− 2.00	0.00
KRT20	− 1.56	0.02
KRT23	− 3.88	0.00
KRT80	− 1.58	0.00
LAMC2	− 1.57	0.00
LFNG	− 1.23	0.00
LGALS1	− 2.44	0.00
LIPG	1.38	0.00
LITAF	− 1.20	0.01
LTBP3	− 1.63	0.00
LTBP4	1.17	0.00
LY6E	− 1.74	0.00
LY6G6D	− 2.03	0.01
LY75	− 1.60	0.00
MAPRE3	− 1.23	0.00
MBOAT2	− 1.03	0.02
MCRIP1	− 1.02	0.00
MDK	− 1.36	0.01
MDM2	1.09	0.00
MELTF	− 1.18	0.01
MGLL	− 1.20	0.00
MIR4435-2HG	− 1.47	0.00
MMP14	− 1.64	0.00
MOCOS	1.13	0.00
MOSPD1	− 1.01	0.00
MUC20	− 1.58	0.00
MYBL2	− 1.06	0.00
NABP1	− 1.05	0.01
NDUFC2	− 1.11	0.00
PDP1	− 1.16	0.00
PEA15	− 1.30	0.00
PFDN4	− 1.20	0.00
PHLDB1	− 1.05	0.05
PLAUR	− 1.18	0.00
PLTP	− 1.53	0.03
PMEPA1	− 2.92	0.00
PML	− 1.03	0.00
POLD4	− 1.06	0.00
PPP1R14D	− 2.05	0.00
PRDX5	− 1.09	0.00
PRR15	− 1.06	0.03
PRSS23	− 1.09	0.02
PSMA7	− 1.00	0.00
PSMB10	− 1.27	0.00
QPCT	− 2.23	0.00
QPRT	− 1.32	0.05
RASL11A	− 1.19	0.04
REG4	2.99	0.00
RGCC	− 2.97	0.00
RGMB	1.49	0.00
RHOD	− 1.22	0.01
ROMO1	− 1.28	0.00
RPL22L1	1.12	0.01
RTFDC1	− 1.04	0.00
S100A11	− 1.65	0.00
S100A2	− 2.87	0.00
S100A4	− 1.55	0.02
SDC4	− 1.30	0.00
SLC20A2	− 1.01	0.00
SLC2A1	− 1.42	0.00
SLC2A4RG	− 1.04	0.00
SLC2A8	− 1.04	0.00
SLC39A5	− 1.70	0.01
SLCO1B3	1.46	0.05
SMAD3	− 1.08	0.00
SMIM22	− 1.19	0.01
SNORA73B	1.00	0.01
SPINK1	− 2.72	0.00
SULT2B1	− 1.60	0.00
SYT7	− 1.55	0.00
TACSTD2	− 2.32	0.00
TCF7	− 1.18	0.00
TGFBI	− 2.28	0.00
TIMP2	− 2.59	0.00
TM4SF1	− 1.78	0.00
TMEM176A	− 2.16	0.00
TMEM176B	− 1.85	0.01
TMEM185A	− 1.07	0.00
TMPRSS4	− 1.35	0.01
TNFRSF14-AS1	1.19	0.02
TNFRSF1B	− 1.44	0.00
TRIB1	− 1.11	0.02
TRIM7	1.08	0.04
TSC22D1	− 1.01	0.01
TUBA4A	− 1.01	0.00
TUBB2A	− 1.49	0.00
TUBE1	1.12	0.00
UCA1	− 1.49	0.03
UCP2	− 1.16	0.02
VAMP8	− 1.10	0.00
VEGFB	− 1.25	0.00
VOPP1	− 1.27	0.00
VSIR	− 1.68	0.00
ZFP90	1.13	0.00
ZMYND8	− 1.07	0.00
EVOLVING-CRC vs STABLE-CRC		
ABCB6	− 1.00	0.04
AHI1	1.04	0.02
ANXA6	− 1.83	0.02
ATOX1	− 1.07	0.01
B2M	− 1.57	0.00
CPNE1	− 1.12	0.00
FAT1	1.09	0.04
GTF3A	− 1.09	0.03
IGFBP6	− 2.01	0.02
IRF1	− 1.05	0.02
LGALS1	− 2.45	0.00
LY6E	− 1.47	0.02
PAM	− 1.27	0.03
PEA15	− 1.12	0.01
PLA2G2A	2.56	0.01
PSMB10	− 1.05	0.02
S100A2	− 1.96	0.00
SDC4	− 1.04	0.05
SLC7A11	1.63	0.01
TGFBI	− 1.50	0.04
TIMP2	− 1.82	0.03
TUBA1A	− 1.71	0.05
TUBB2A	− 1.55	0.03
TUBE1	1.27	0.01
VEGFA	1.08	0.05

## Discussion

In the past decade, it has become clear that most human tumors are highly molecularly heterogeneous, and this affects prognosis and the emergence of therapeutic resistance [40]. How tumor-specific somatic variations can lead to distinct neoantigen profiles and ultimately to immune surveillance has also been partially elucidated. The number of neoantigens depends on several factors. For example, lung cancers associated with smoking habits have high levels of mutations [41, 42], whereas the development of skin melanomas is correlated with UV light-mediated mutagenicity [43]. Both smoking and UV exposure occur during defined periods and their mutagenicity is transient, leading to high—but relatively stable—mutational profiles [44, 45]. Another class of tumors with high mutational burden is characterized not by exposure to external carcinogens, but rather by the intrinsic inability of tumor cells to efficiently repair DNA. The latter is due to epigenetic or genetic alterations in key effectors of DNA repair pathways, rather than acute or chronic carcinogen exposure. In this work, we used CRC as a model system to understand whether and to what extent alterations of DNA repair pathway components modulate neoantigen profiles over time in vitro and in vivo. Tumors carrying alterations affecting DNA repair genes maintained their molecular characteristics over time, and in most instances, the functional consequence of those alterations is continuous and propagated at every generation. An exception was represented by two *POLE* mutant CRC cell lines (HROC69 and HCC2998) which despite having high mutational burden did not appreciably evolve over time. The reason(s) for this phenotype is presently unclear. Interestingly, these two *POLE* mutant cells that evolved poorly over time had less marked mutational signatures, possibly suggesting that, in these models, polymerase defects may undergo some form of functional compensation.

The longitudinal analysis of cell and PDX models highlighted several aspects. For example, MSI- and POLE-mutated tumors tended to acquire SNV or short insertions/deletions over time. These alterations can lead to novel putative neoantigens which potentially trigger the host immune system. In addition to well-known DDR genes (*MLH1, MSH2, MSH6, PMS2, POLE*), our study indicate that other genes involved in DNA repair pathway may lead to accumulations of mutations possibly translating in novel epitopes. *EXO1* and *MUTYH* are two of such examples. Profiling of these genes in the clinical setting may help to intercept tumors not classified as unstable or with hypermutator phenotype but nevertheless continuously evolving and accumulating mutations.

Our analysis suggests that in parallel to mutation gains, loss of variants also occurs during cell propagation. Our data indicate that in hypermutated CRCs, including MSI- and *POLE*-mutated models expanded in vitro, these events are mainly confined to subclones. A limitation of this study is that longitudinal characterization of lost and gained mutations in vitro could be influenced by sampling of cell populations during cell passaging. We also report that in the propagation of PDXs, possibly due to selection imposed by the microenvironment, not only subclonal but also clonal variants emerge de novo over time. Based on these results, we speculate that in CRC patients with DNA repair defects metastatic seeding or therapeutic debulking can lead to the emergence of new subsets of clonal neoantigens. This could have implications for the development of therapies relying on the presence of clonal neoantigens, such as ICP, CAR-T, and vaccines.

Both cell lines and PDXs have been widely employed to test anticancer compounds [46–48]; however, experimental reproducibility has occasionally been questioned [49, 50]. The molecular evolvability that we find to occur during serial passaging of cells and PDXs may partly account for the discrepant results obtained with these models [51–53].

A limitation of the present study is that it examined the evolution of cell lines and xenografts but cannot address the impact of the immune system in the evolutionary dynamics due to intrinsic limitations of the models we used.

Our data indicate that alterations in DNA repair genes facilitate the acquisition of neoantigens. These novel putative epitopes can be recognized by the immune system. Accordingly, we confirm that CRCs with high number of mutations (hypermutated CRCs) selectively downregulate components of the neoantigen presentation process, such as *B2M*, thus restricting the ability of the host immune system to detect them. Our results further suggest that non-hypermutated CRCs, that display fast evolving mutational and antigen profiles, also show downregulation of components implicated in neoantigen presentation. The differences in expression of molecules involved in immune functions we observed in the CRC models could have originated from adaption previously experienced in the patient as a mechanism of escape from negative pressure of the immune system related to the elevated neoantigens’ production rate.

## Conclusions

In summary, we identified and functionally highlighted CRC subsets characterized by slow and fast genome evolvability. CRCs carrying alterations in genes involved in DNA repair (including *MLH1*, *MSH2*, *MSH6*, *MUTYH*, *EXO1*, and *POLE*) display dynamic neoantigen patterns that fluctuate over time. Furthermore, we find that in CRC cells and patient-derived tumor xenografts, DNA repair defects leading to high mutational burden and neoantigen evolvability are associated with inactivation or downregulation of antigen-presentation functions. Longitudinal monitoring of the neoantigen landscape of CRC and other tumor types may have clinical implications. While tracking time-dependent neoantigen evolution in the tissue of cancer patients might be difficult or impossible to achieve, monitoring predicted neoantigens in circulating tumor DNA is already within reach. Accordingly, longitudinal liquid biopsies could be deployed to assess whether and how time and/or therapeutic regimens affect the mutational burden and the neoantigen profiles in individual patients. Neoantigen clonality profiles could be valuable to develop specific vaccines and deploy immunomodulatory molecules in the context of precision oncology.

## Additional files

Additional file 1: Supplementary figures S1-S15. (PDF 2606 kb)

Additional file 2: Single nucleotide variants and frameshifts of 64 CRC cell lines at T0. (XLSX 9992 kb)

## Data Availability

Detailed mutational characterization of the 64 CRC cell lines is available in Additional file 2. NGS data from cell lines used in the current study are available in the European Nucleotide Archive (ENA) with the following accession code PRJEB33045.

## References

[1] Overman MJ, McDermott R, Leach JL, Lonardi S, Lenz HJ, Morse MA (2017). Nivolumab in patients with metastatic DNA mismatch repair-deficient or microsatellite instability-high colorectal cancer (CheckMate 142): an open-label, multicentre, phase 2 study. Lancet Oncol.

[2] Gibney GT, Weiner LM, Atkins MB (2016). Predictive biomarkers for checkpoint inhibitor-based immunotherapy. Lancet Oncol..

[3] Ribas A, Wolchok JD (2018). Cancer immunotherapy using checkpoint blockade. Science..

[4] Cristescu Razvan, Mogg Robin, Ayers Mark, Albright Andrew, Murphy Erin, Yearley Jennifer, Sher Xinwei, Liu Xiao Qiao, Lu Hongchao, Nebozhyn Michael, Zhang Chunsheng, Lunceford Jared K., Joe Andrew, Cheng Jonathan, Webber Andrea L., Ibrahim Nageatte, Plimack Elizabeth R., Ott Patrick A., Seiwert Tanguy Y., Ribas Antoni, McClanahan Terrill K., Tomassini Joanne E., Loboda Andrey, Kaufman David (2018). Pan-tumor genomic biomarkers for PD-1 checkpoint blockade–based immunotherapy. Science.

[5] Lee CH, Yelensky R, Jooss K, Chan TA (2018). Update on tumor neoantigens and their utility: why it is good to be different. Trends Immunol.

[6] Samstein RM, Lee CH, Shoushtari AN, Hellmann MD, Shen R, Janjigian YY (2019). Tumor mutational load predicts survival after immunotherapy across multiple cancer types. Nat Genet.

[7] Shlien A, Campbell BB, de Borja R, Alexandrov LB, Merico D, Wedge D (2015). Combined hereditary and somatic mutations of replication error repair genes result in rapid onset of ultra-hypermutated cancers. Nat Genet.

[8] Turajlic S, Litchfield K, Xu H, Rosenthal R, McGranahan N, Reading JL (2017). Insertion-and-deletion-derived tumour-specific neoantigens and the immunogenic phenotype: a pan-cancer analysis. Lancet Oncol..

[9] Germano G, Lamba S, Rospo G, Barault L, Magrì A, Maione F (2017). Inactivation of DNA repair triggers neoantigen generation and impairs tumour growth. Nature..

[10] Conway T, Wazny J, Bromage A, Tymms M, Sooraj D, Williams ED (2012). Xenome--a tool for classifying reads from xenograft samples. Bioinformatics..

[11] Li H, Durbin R (2010). Fast and accurate long-read alignment with Burrows-Wheeler transform. Bioinformatics..

[12] Li H, Handsaker B, Wysoker A, Fennell T, Ruan J, Homer N (2009). The sequence alignment/map format and SAMtools. Bioinformatics..

[13] Siravegna G, Mussolin B, Buscarino M, Corti G, Cassingena A, Crisafulli G, et al. Clonal evolution and resistance to EGFR blockade in the blood of colorectal cancer patients. Nat Med. 2015;21(7):795–801. 10.1038/nm.3870 PMC4868598 26030179

[14] Germano Giovanni, Amirouchene-Angelozzi Nabil, Rospo Giuseppe, Bardelli Alberto (2018). The Clinical Impact of the Genomic Landscape of Mismatch Repair–Deficient Cancers. Cancer Discovery.

[15] Szolek A, Schubert B, Mohr C, Sturm M, Feldhahn M, Kohlbacher O (2014). OptiType: precision HLA typing from next-generation sequencing data. Bioinformatics..

[16] Andreatta M, Nielsen M (2016). Gapped sequence alignment using artificial neural networks: application to the MHC class I system. Bioinformatics..

[17] Díaz-Gay M, Vila-Casadesús M, Franch-Expósito S, Hernández-Illán E, Lozano JJ, Castellví-Bel S (2018). Mutational Signatures in Cancer (MuSiCa): a web application to implement mutational signatures analysis in cancer samples. BMC Bioinformatics.

[18] Alexandrov LB, Nik-Zainal S, Wedge DC, Aparicio SA, Behjati S, Biankin AV (2013). Signatures of mutational processes in human cancer. Nature..

[19] Hirsch HR, Engelberg J (1965). Determination of the cell doubling-time distribution from culture growth-rate data. J Theor Biol.

[20] Wang K, Singh D, Zeng Z, Coleman SJ, Huang Y, Savich GL (2010). MapSplice: accurate mapping of RNA-seq reads for splice junction discovery. Nucleic Acids Res.

[21] Li B, Dewey CN (2011). RSEM: accurate transcript quantification from RNA-Seq data with or without a reference genome. BMC Bioinformatics..

[22] Love MI, Huber W, Anders S (2014). Moderated estimation of fold change and dispersion for RNA-seq data with DESeq2. Genome Biol.

[23] Reimand J, Arak T, Adler P, Kolberg L, Reisberg S, Peterson H (2016). g:Profiler-a web server for functional interpretation of gene lists (2016 update). Nucleic Acids Res.

[24] Galimi F, Torti D, Sassi F, Isella C, Corà D, Gastaldi S (2011). Genetic and expression analysis of MET, MACC1, and HGF in metastatic colorectal cancer: response to met inhibition in patient xenografts and pathologic correlations. Clin Cancer Res.

[25] Siravegna G, Lazzari L, Crisafulli G, Sartore-Bianchi A, Mussolin B, Cassingena A (2018). Radiologic and genomic evolution of individual metastases during HER2 blockade in colorectal cancer. Cancer Cell.

[26] Corti Giorgio, Bartolini Alice, Crisafulli Giovanni, Novara Luca, Rospo Giuseppe, Montone Monica, Negrino Carola, Mussolin Benedetta, Buscarino Michela, Isella Claudio, Barault Ludovic, Siravegna Giulia, Siena Salvatore, Marsoni Silvia, Di Nicolantonio Federica, Medico Enzo, Bardelli Alberto (2019). A Genomic Analysis Workflow for Colorectal Cancer Precision Oncology. Clinical Colorectal Cancer.

[27] Network CGA (2012). Comprehensive molecular characterization of human colon and rectal cancer. Nature..

[28] Goellner EM, Putnam CD, Kolodner RD (2015). Exonuclease 1-dependent and independent mismatch repair. DNA Repair (Amst).

[29] Boiteux S, Coste F, Castaing B (2017). Repair of 8-oxo-7,8-dihydroguanine in prokaryotic and eukaryotic cells: properties and biological roles of the Fpg and OGG1 DNA N-glycosylases. Free Radic Biol Med.

[30] Dallosso AR, Dolwani S, Jones N, Jones S, Colley J, Maynard J (2008). Inherited predisposition to colorectal adenomas caused by multiple rare alleles of MUTYH but not OGG1, NUDT1, NTH1 or NEIL 1, 2 or 3. Gut.

[31] Sim NL, Kumar P, Hu J, Henikoff S, Schneider G, Ng PC (2012). SIFT web server: predicting effects of amino acid substitutions on proteins. Nucleic Acids Res.

[32] Adzhubei IA, Schmidt S, Peshkin L, Ramensky VE, Gerasimova A, Bork P (2010). A method and server for predicting damaging missense mutations. Nat Methods.

[33] Viel A, Bruselles A, Meccia E, Fornasarig M, Quaia M, Canzonieri V (2017). A specific mutational signature associated with DNA 8-oxoguanine persistence in MUTYH-defective colorectal cancer. EBioMedicine..

[34] Gajewski TF, Schreiber H, Fu YX (2013). Innate and adaptive immune cells in the tumor microenvironment. Nat Immunol.

[35] Bertotti A, Papp E, Jones S, Adleff V, Anagnostou V, Lupo B (2015). The genomic landscape of response to EGFR blockade in colorectal cancer. Nature..

[36] Guinney J, Dienstmann R, Wang X, de Reyniès A, Schlicker A, Soneson C (2015). The consensus molecular subtypes of colorectal cancer. Nat Med.

[37] Isella C, Terrasi A, Bellomo SE, Petti C, Galatola G, Muratore A (2015). Stromal contribution to the colorectal cancer transcriptome. Nat Genet.

[38] Sinicrope FA, Rego RL, Halling KC, Foster N, Sargent DJ, La Plant B (2006). Prognostic impact of microsatellite instability and DNA ploidy in human colon carcinoma patients. Gastroenterology..

[39] Tian S, Roepman P, Popovici V, Michaut M, Majewski I, Salazar R (2012). A robust genomic signature for the detection of colorectal cancer patients with microsatellite instability phenotype and high mutation frequency. J Pathol.

[40] Dagogo-Jack I, Shaw AT (2018). Tumour heterogeneity and resistance to cancer therapies. Nat Rev Clin Oncol.

[41] Govindan R, Ding L, Griffith M, Subramanian J, Dees ND, Kanchi KL (2012). Genomic landscape of non-small cell lung cancer in smokers and never-smokers. Cell..

[42] Alexandrov LB, Ju YS, Haase K, Van Loo P, Martincorena I, Nik-Zainal S (2016). Mutational signatures associated with tobacco smoking in human cancer. Science..

[43] Hodis E, Watson IR, Kryukov GV, Arold ST, Imielinski M, Theurillat JP (2012). A landscape of driver mutations in melanoma. Cell..

[44] Network CGAR (2014). Comprehensive molecular profiling of lung adenocarcinoma. Nature..

[45] Network CGA (2015). Genomic classification of cutaneous melanoma. Cell..

[46] Yang W, Soares J, Greninger P, Edelman EJ, Lightfoot H, Forbes S (2013). Genomics of drug sensitivity in cancer (GDSC): a resource for therapeutic biomarker discovery in cancer cells. Nucleic Acids Res.

[47] Garnett MJ, Edelman EJ, Heidorn SJ, Greenman CD, Dastur A, Lau KW (2012). Systematic identification of genomic markers of drug sensitivity in cancer cells. Nature..

[48] Barretina J, Caponigro G, Stransky N, Venkatesan K, Margolin AA, Kim S (2012). The Cancer Cell Line Encyclopedia enables predictive modelling of anticancer drug sensitivity. Nature..

[49] Iorio F, Knijnenburg TA, Vis DJ, Bignell GR, Menden MP, Schubert M (2016). A landscape of pharmacogenomic interactions in cancer. Cell..

[50] Vanden Heuvel JP, Bullenkamp J, Biology RPC. Registered report: systematic identification of genomic markers of drug sensitivity in cancer cells. Elife. 2016;5:1–19. 10.7554/eLife.13620 PMC4919108 27336789

[51] Liu Y, Mi Y, Mueller T, Kreibich S, Williams EG, Van Drogen A (2019). Multi-omic measurements of heterogeneity in HeLa cells across laboratories. Nat Biotechnol.

[52] Ben-David U, Siranosian B, Ha G, Tang H, Oren Y, Hinohara K (2018). Genetic and transcriptional evolution alters cancer cell line drug response. Nature..

[53] Ben-David U, Ha G, Tseng YY, Greenwald NF, Oh C, Shih J (2017). Patient-derived xenografts undergo mouse-specific tumor evolution. Nat Genet.

