# Supplementary material for: Evolving neoantigen profiles in colorectal cancers with DNA repair defects
Source: Genome Med. 2019 Jun 28;11:42. doi: 10.1186/s13073-019-0654-6 (PMC6599263; doi:10.1186/s13073-019-0654-6)
Supplement: Supplementary file 1 — Supplementary figures S1-S15. (PDF 2606 kb) [file 13073_2019_654_MOESM1_ESM.pdf]

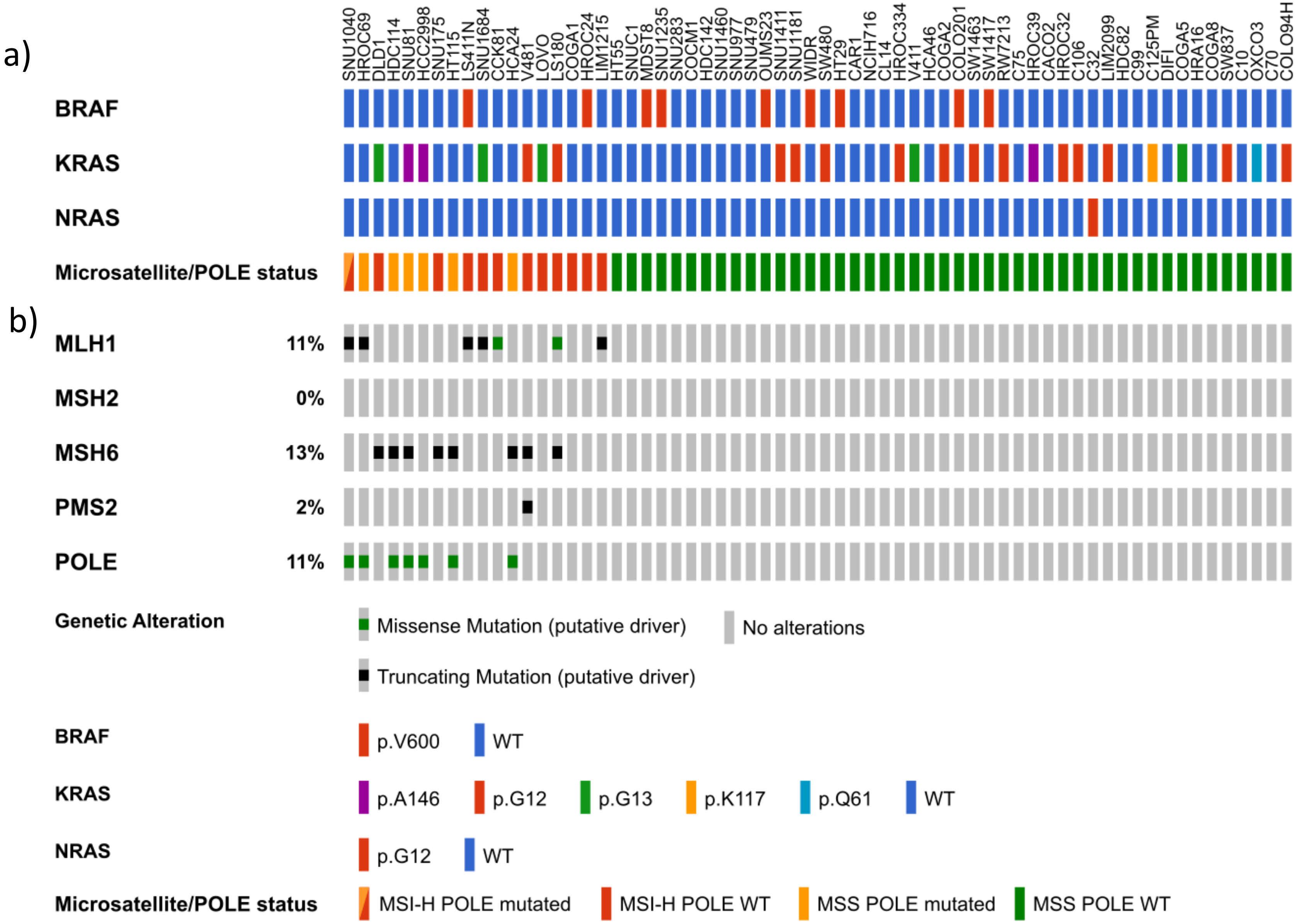

Fig. S1 Genomic features of 64 CRC cell lines. Molecular characterization of the indicated CRC models at T0 using cbioportal oncoprint graphic representation. a) Schematic diagram showing *BRAF*, *KRAS*, *NRAS* and Microsatellite/*POLE* status of CRC cell lines. b) Schematic diagram showing genetic alterations in MMR genes and DNA proofreading polymerase *POLE* in CRC cell lines.

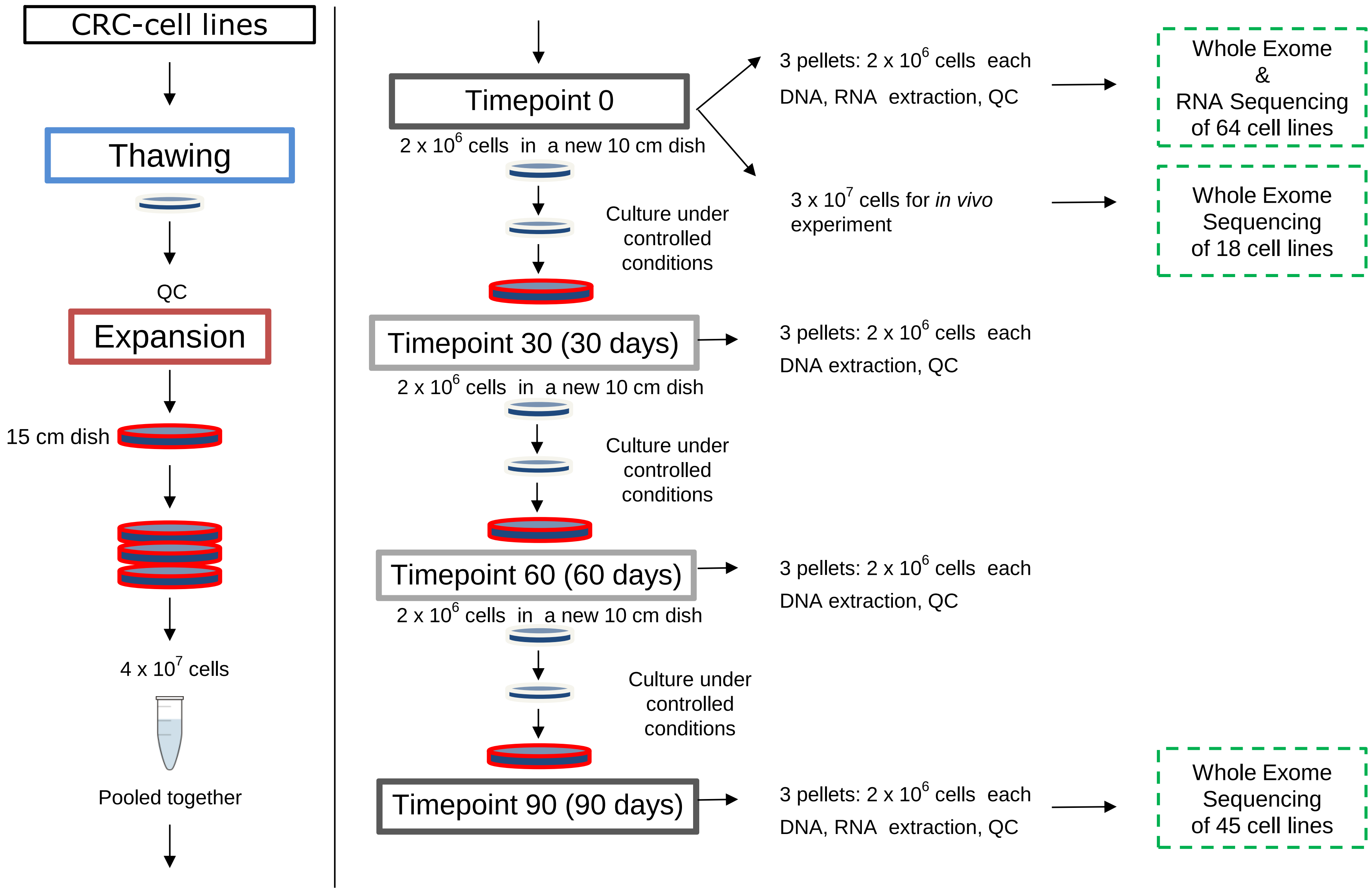

Fig. S2 Outline of the experimental workflow to assess evolution of CRC cell lines *in vitro* and *in vivo*. CRC cell lines were thawed and kept in culture for 90 days. WES and RNAseq were performed at the beginning of the experiment (T0) for 64 cell lines. Forty-five samples collected at T90 were also subjected to WES. In a few instances at T0 an equal number of cells was injected in two immunodeficient mice. When tumours reached approximately a volume of 1000m^3 in size, they were excised and sequenced.

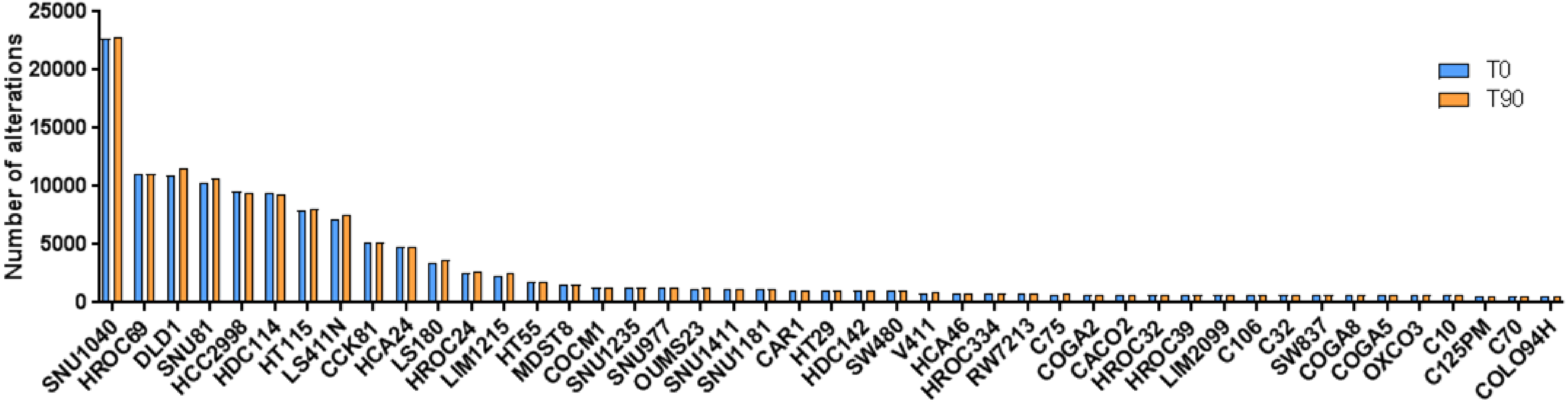

Fig. S3 Genetic alterations in CRC cell lines at T0 and T90. Bar chart showing the number of alterations at the beginning of experiment (T0) and after 90 days of culture (T90). All the variants are called against the hg38 reference.

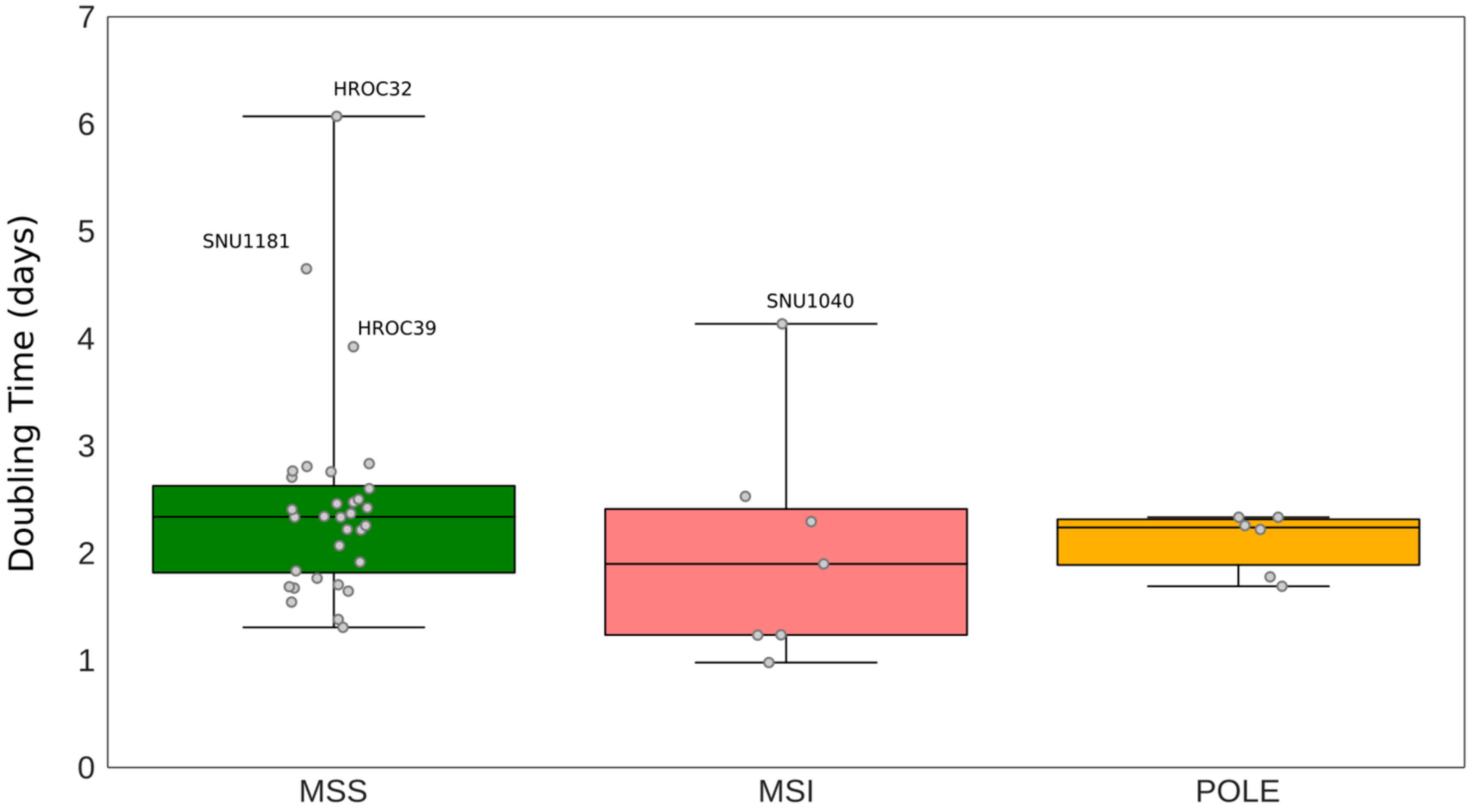

Fig. S4 Doubling time of MSS, MSI and POLE mutated cell lines. Cell doubling time in MSS, MSI and MSS *POLE* mutant cells. The number of days per group is shown. The centre line of each box plot indicates the median.

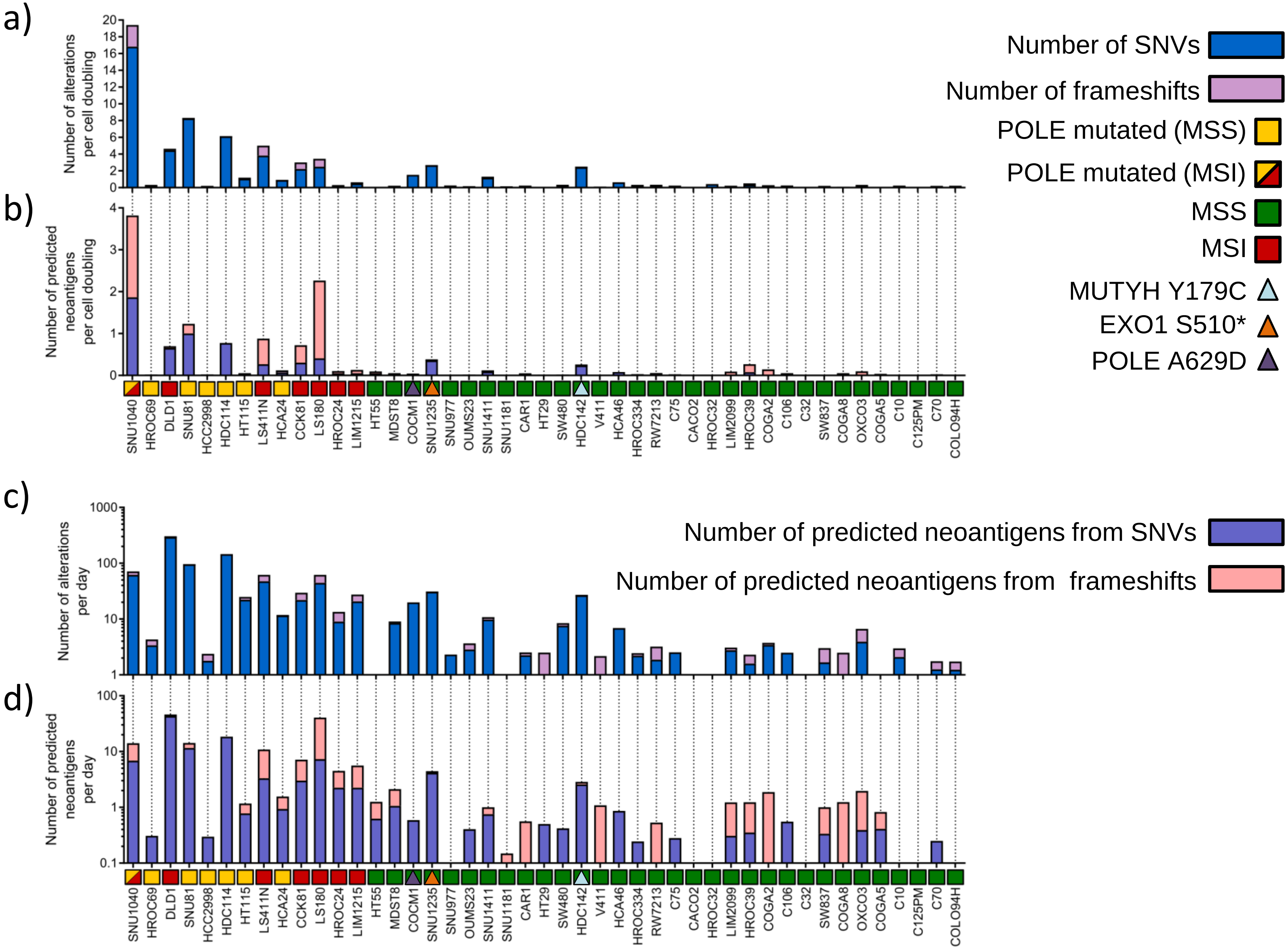

Fig. S5 *In vitro* evolution of mutational landscape in CRC cell line normalized to the doubling time. Mutational characterization of CRC cells after 90 days of culture (T90) normalized to the doubling time. a) The bar chart shows the number of new alterations acquired at T90 (absent at T0) normalized to the cell doubling time. b) The number of predicted neoantigens per cell doubling (see Methods) is shown. Each bar represents putative neoepitopes derived from SNVs and frameshifts. c) The bar chart shows the number of new alterations per day acquired at T90 (absent at T0). d) The number of predicted neoantigens acquired per day (see Methods) is listed. Each bar represents putative neoepitopes derived from SNVs and frameshifts.

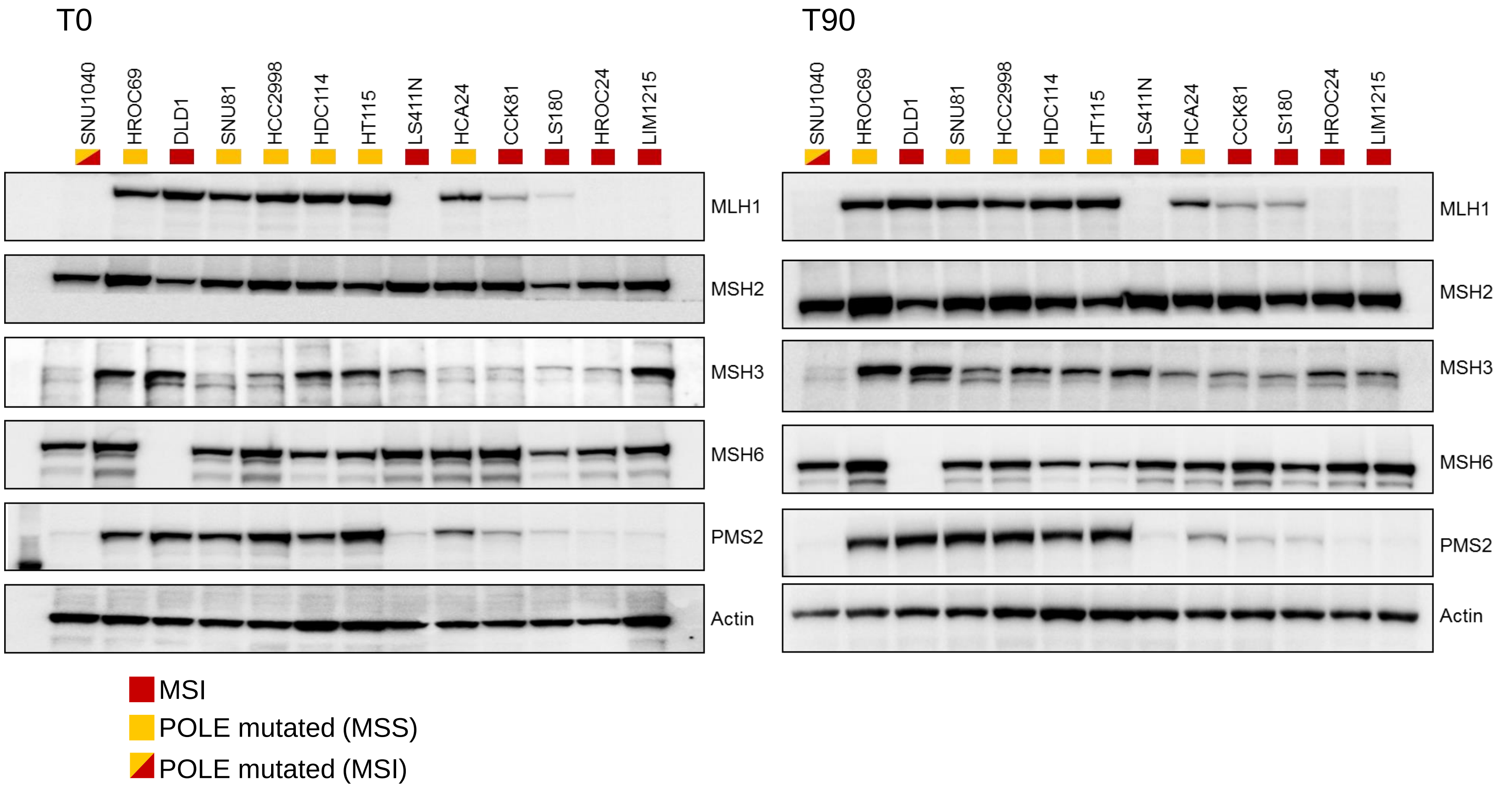

Fig. S6 MMR proteins expression in cell models. Western blot analysis on hypermutated samples (MSI and/or POLE mutant cell lines) to assess the status of the MMR proteins at Time 0 (T0) and Time 90 (T90).

POLE A629D

Species	Gene	Protein
H.sapiens	POLE	NP_006222.2
P.troglodytes	POLE	XP_522564.4
C.lupus	POLE	XP_543348.3
B.taurus	POLE	NP_001178358.1
M.musculus	Pole	NP_035262.2
R.norvegicus	Pole	NP_001100622.2
G.gallus	POLE	XP_004934482.1
D.rerio	pole	NP_001121995.1
D.melanogaster	DNApol-epsilon	NP_524462.2
A.gambiae	AgaP_AGAP004615	XP_315205.4
C.elegans	F33H2.5	NP_493616.1
S.cerevisiae	POL2	NP_014137.1
K.lactis	KLLA0C02585g	XP_452312.1
E.gossypii	AGOS_AFR657C	NP_986205.2
S.pombe	cdc20	NP_596354.1
318829	MGG_03850	XP_003720041.1
N.crassa	NCU04548	XP_955939.2
A.thaliana	TIL2	NP_180280.2
A.thaliana	TIL1	NP_172303.5
O.sativa	Os02g0511900	NP_001046939.2
X.tropicalis	pole	XP_004910692.1

```
NP_006222.2    603 IKSKLASLKDVPSRIECPLIYHLDVGAMYPNIILTNRLQPSAMVDEATCA 652
XP_522564.4    659 IKSKLASLKDVPSRIECPLIYHLDVGAMYPNIILTNRLQPSAMVDEATCA 708
XP_543348.3    560 IKTKLTSLKDVPNRIECPLIYHLDVGAMYPNIILTNRLQPSAVVDEATCA 609
NP_001178358.1 603 IKTKLTSLKDVPNRIECPLIYHLDVGAMYPNIILTNRLQPSAMVDEATCA 652
NP_035262.2    603 IKTKLTSLKDVPNRIECPLIYHLDVGAMYPNIILTNRLQPSAIVDEATCA 652
NP_001100622.2 603 IKTKLTSLKDVPNRIECPLIYHLDVGAMYPNIILTNRLQPSAIVDEATCA 652
XP_004934482.1 628 IKVKLNSLKDVPNRIECPLIYHLDVGAMYPNIILTNRLQPSAMVDEATCA 677
NP_001121995.1 602 IKRKLISLKEVPNRIECPLIYHLDVGAMYPNIILTNRLQPSAMVDEATCA 651
NP_524462.2    601 IVQGLQGLHDIPNRLEQPVIYHLDVGAMYPNIILTNRLQPSAMVSDLDCA 650
XP_315205.4    597 ILSALQQLYDIPTRLEQPVIYHLDVGAMYPNIILTNRLQPSSMVNDADCA 646
NP_493616.1    588 VQDAFDGMINVPTRLENPRIYHLDVGAMYPNIILTNRLQPCAMVTEEICM 637
NP_014137.1    617 ITQKLLELKENNIRNELPLIYHVDVASMYPNIMTTNRLQPDSIKAERDCA 666
XP_452312.1    594 ITAQLTDLKINNKRNELPLIYHVDVASMYPNIMTTNRLQPDSMKDEKDCA 643
NP_986205.2    591 ITEQLQELKMNNKRKELPLIYHVDVASMYPNIMTTNRLQPDSMKTERDCA 640
NP_596354.1    603 ILKKLSDLRDRPKRSEKPRIYHLDVASMYPNIMITNRLQPDSVKDESFCA 652
XP_003720041.1 621 IKAKLLALRDTPNRQERPLIYHLDVASMYPNIMTTNRLQPDSMISEADCA 670
XP_955939.2    621 ITARLMNLKETPNRLERPLIYHLDVASMYPNIMTTNRLQPDSMISESTCA 670
NP_180280.2    567 IREKLEKLRDDPIREEGPLIYHLDVAAMYPNIILTNRLQPPSIVTDEVCT 616
NP_172303.5    575 IKEKLEKLRDDPIREEGPLIYHLDVAAMYPNIILTNRLQPPSIVTDEICT 624
NP_001046939.2 606 IKQKLVSLRDHPIREECPLIYHLDVAAMYPNIILTNRLQPPSIVTDVDCT 655
XP_004910692.1 601 IKKKLNSLKEVPNRIECPLIYHLDVGAMYPNIILTNRLQPSAMVDEVTCA 650
```

Fig. S7 POLE protein sequence alignment across species. The yellow box indicates alanine (A) 629, which is highly conserved across species but is mutated to aspartic acid (D) in the COCM1 cell line.

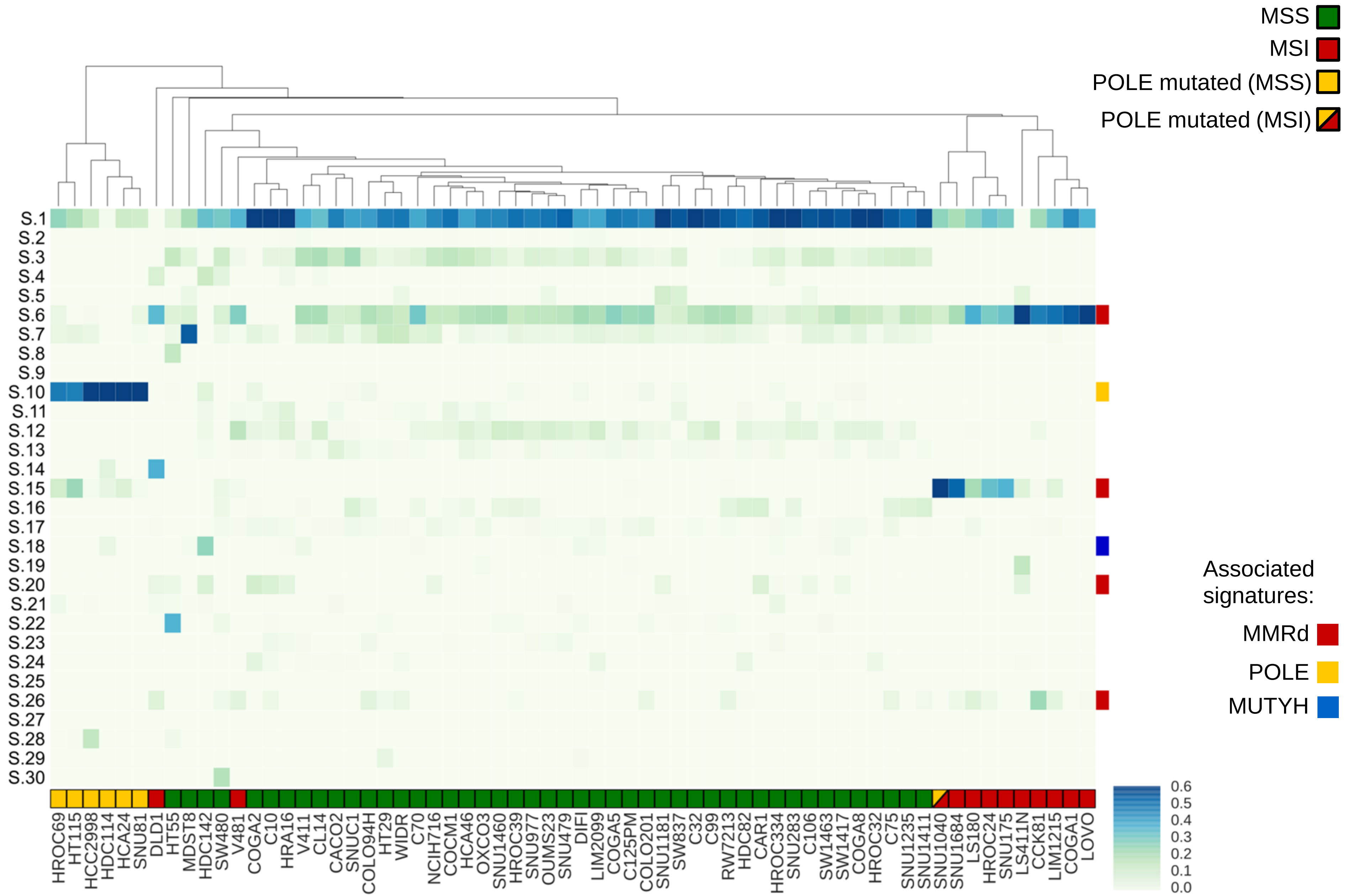

Fig. S8 Analysis of mutational signatures in CRC cell lines. Heatmap and clustering of 30 cancer associated signatures across 64 CRC cell lines at T0. Signatures associated with MMR-deficiency (6, 15, 20 and 26), POLE mutations (10) and MUTYH-associated polyposis (18) are highlighted. Analysis and clustering were performed as reported in Methods.

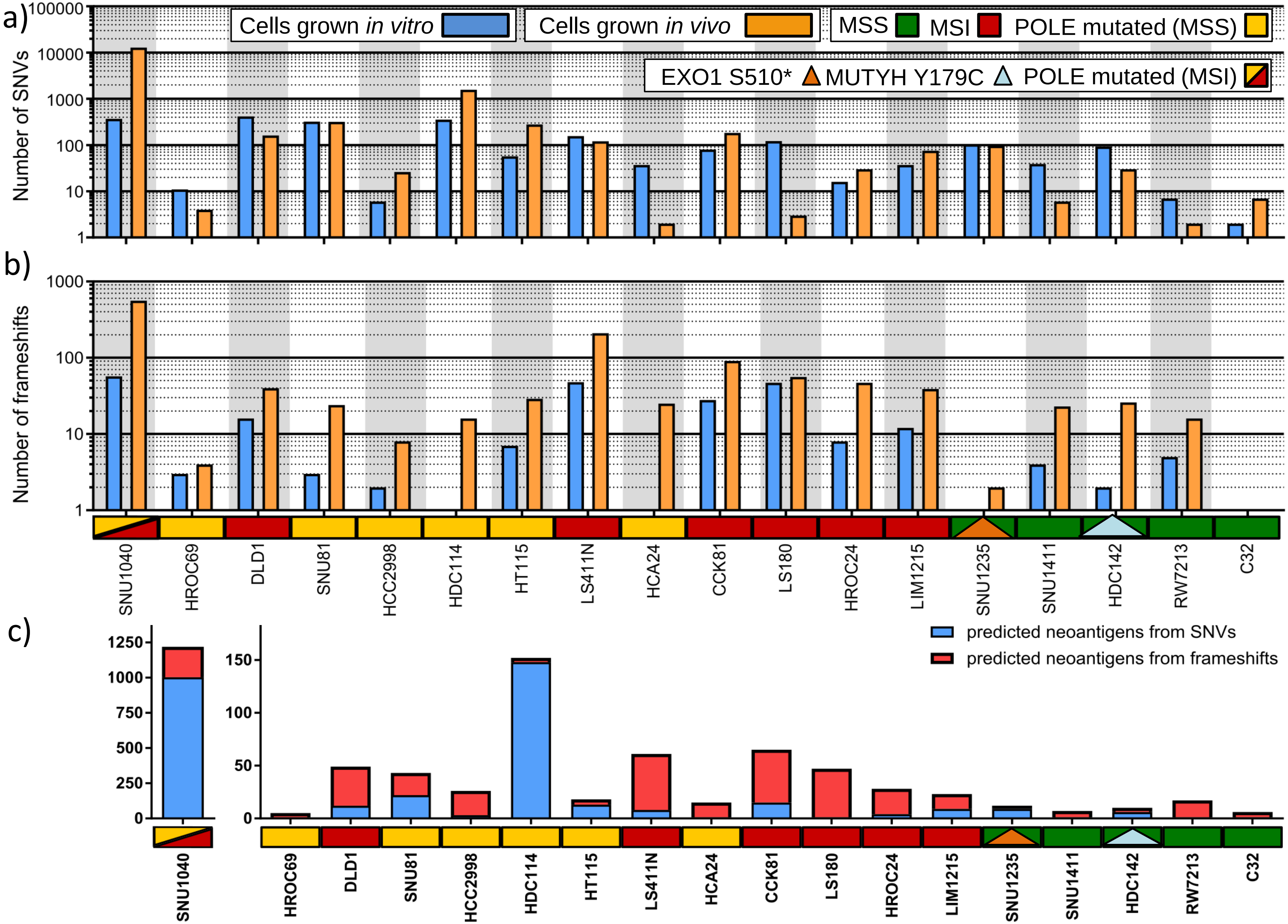

Fig. S9 Comparison of mutational profiles in cell lines grown *in vitro* and *in vivo*. Alterations acquired by the indicated cell lines after 90 days in cell culture and upon transplantation in mice. a) Number of SNVs/Mb acquired *in vitro* (blue) and *in vivo* (orange). b) Number of frameshifts acquired *in vitro* (blue) and *in vivo* (orange). c) Number of predicted neoantigens in CRC cell lines after injection in mice (See Method for details).

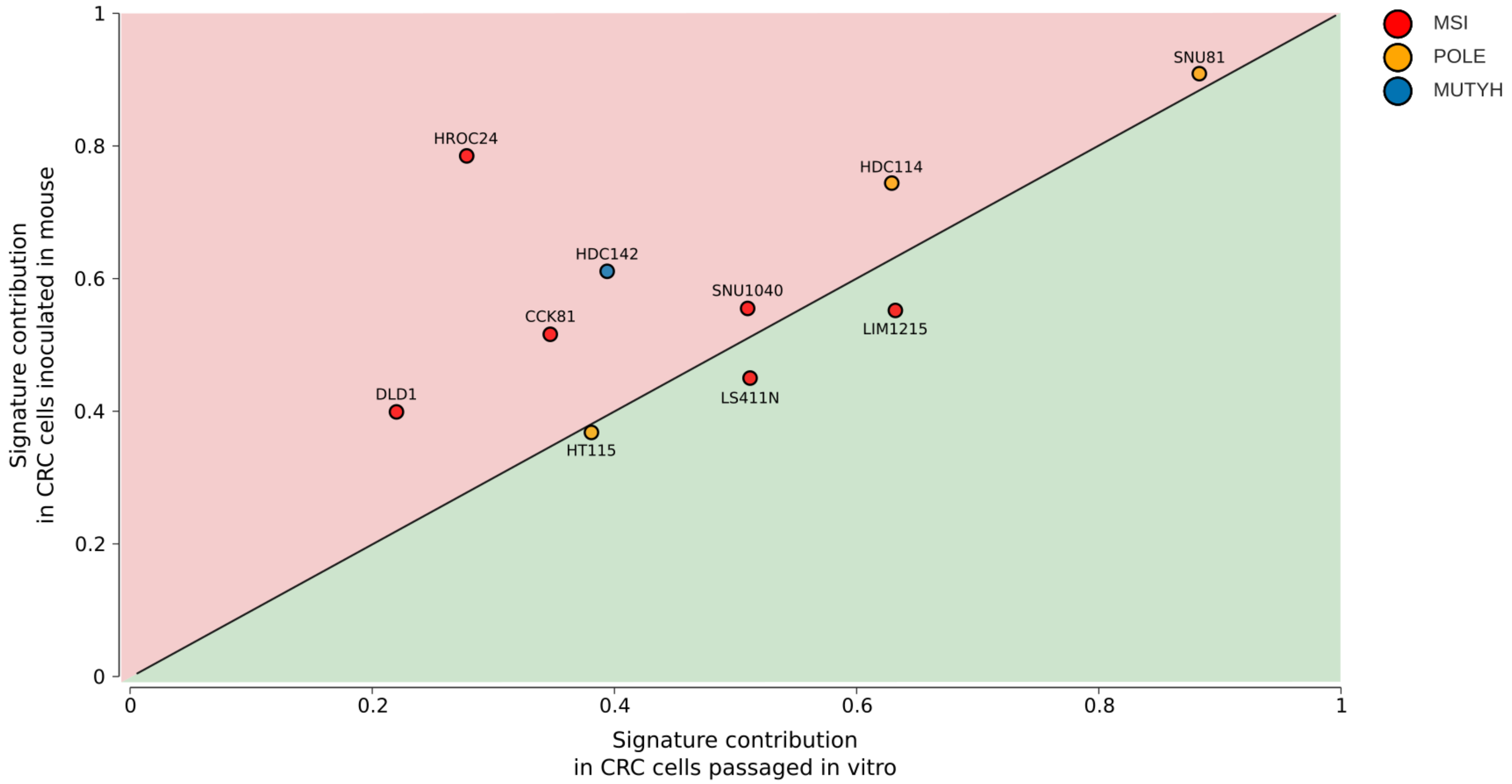

Fig. S10 Comparison of signatures contribution between CRCs models propagated *in vitro* and *in vivo*. The contributions of the associated signatures based on the molecular status detected *in vitro* and *in vivo* were compared in each model. For the MSI samples we compared the maximum contribution provided by signatures 6, 15, 20 or 26 (linked to MMRd). For the MSS POLE mutant cells, contribution was from signature number 10. For the cell line carrying MUTYH biallelic alterations contribution was from signature 18.

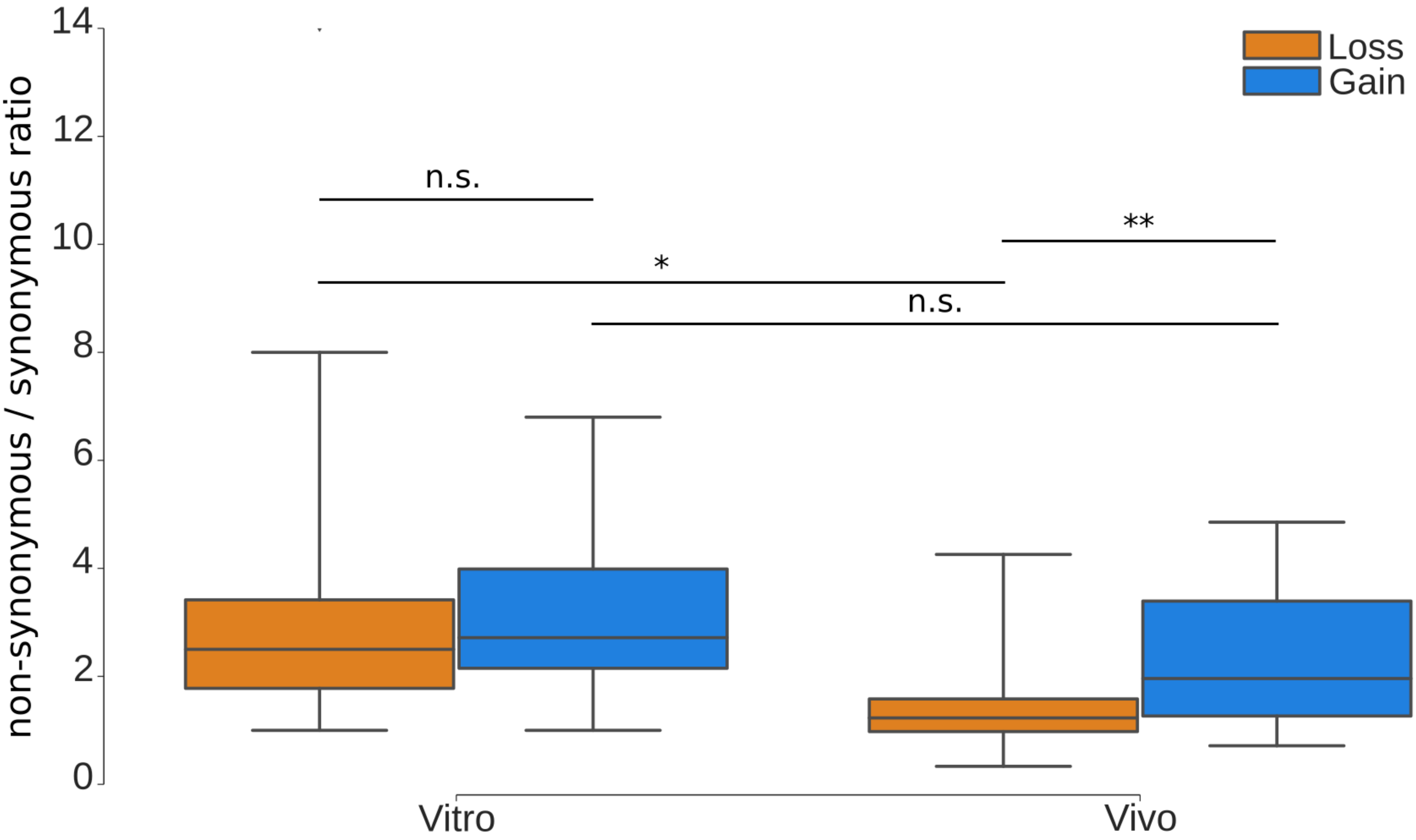

Fig. S11 Comparison of non-synonymous over synonymous mutations ratio in CRC cells expanded *in vitro* and upon transplantation in mice. Characterization of ratios between non-synonymous and synonymous variants in 19 CRC cells passaged *in vitro* and expanded *in vivo.* $P < 0.05$ in lost variants ratios comparing *Vitro* and *Vivo* groups. $P < 0.005$ in lost *vs* gained mutations' ratios *in vivo.*

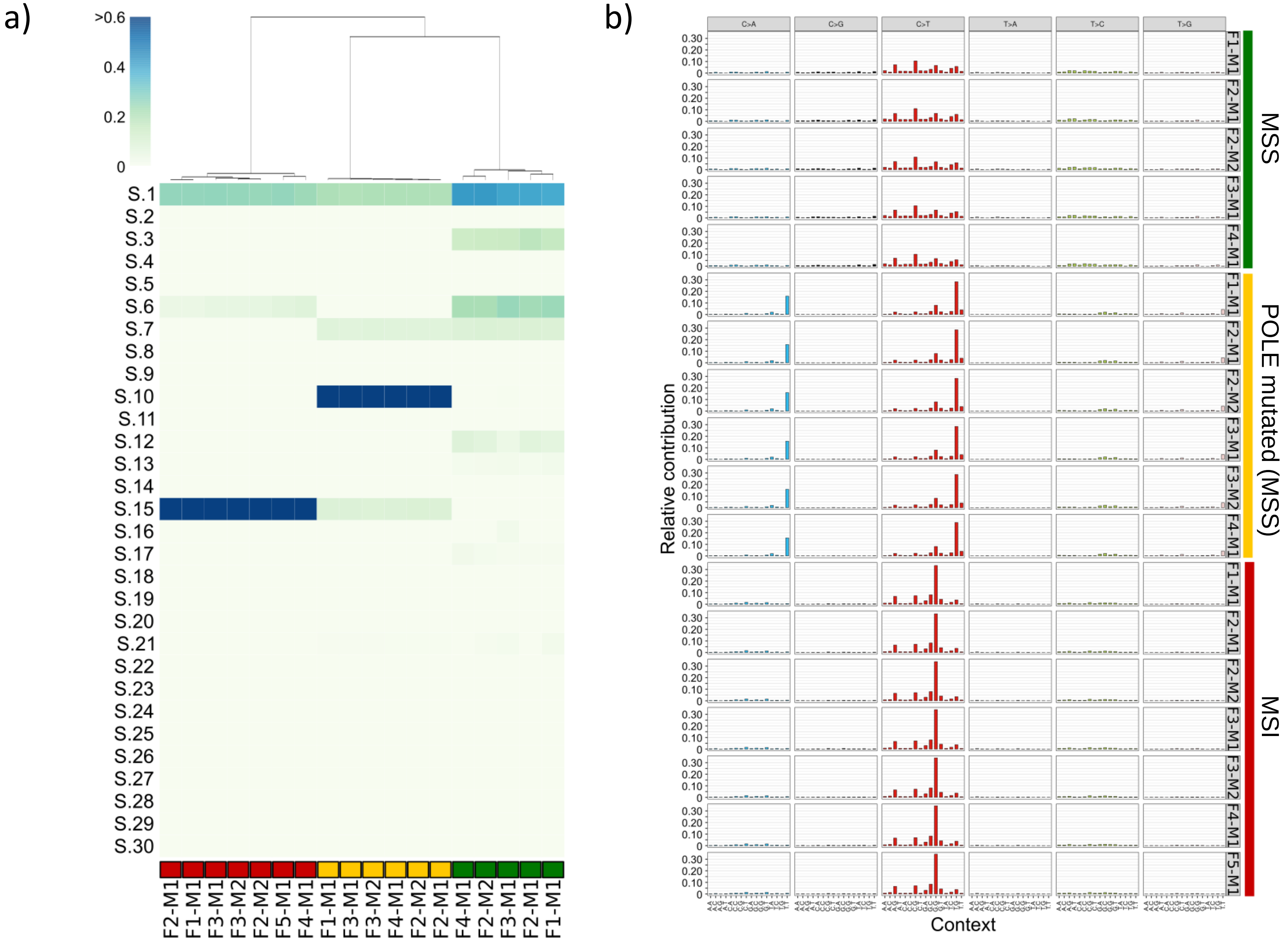

Fig. S12 Analysis of mutational signatures in patient-derived xenograft. Contributions of 30 cancer associated signatures in patient-derived CRC xenografts. (a) Clustered heatmap according to signatures contribution in the indicated PDXs. (b) Signature profiles in PDXs using the six substitution subtypes: C>A, C>G, C>T, T>A, T>C, and T>G. Analysis and clustering were performed as reported in Methods.

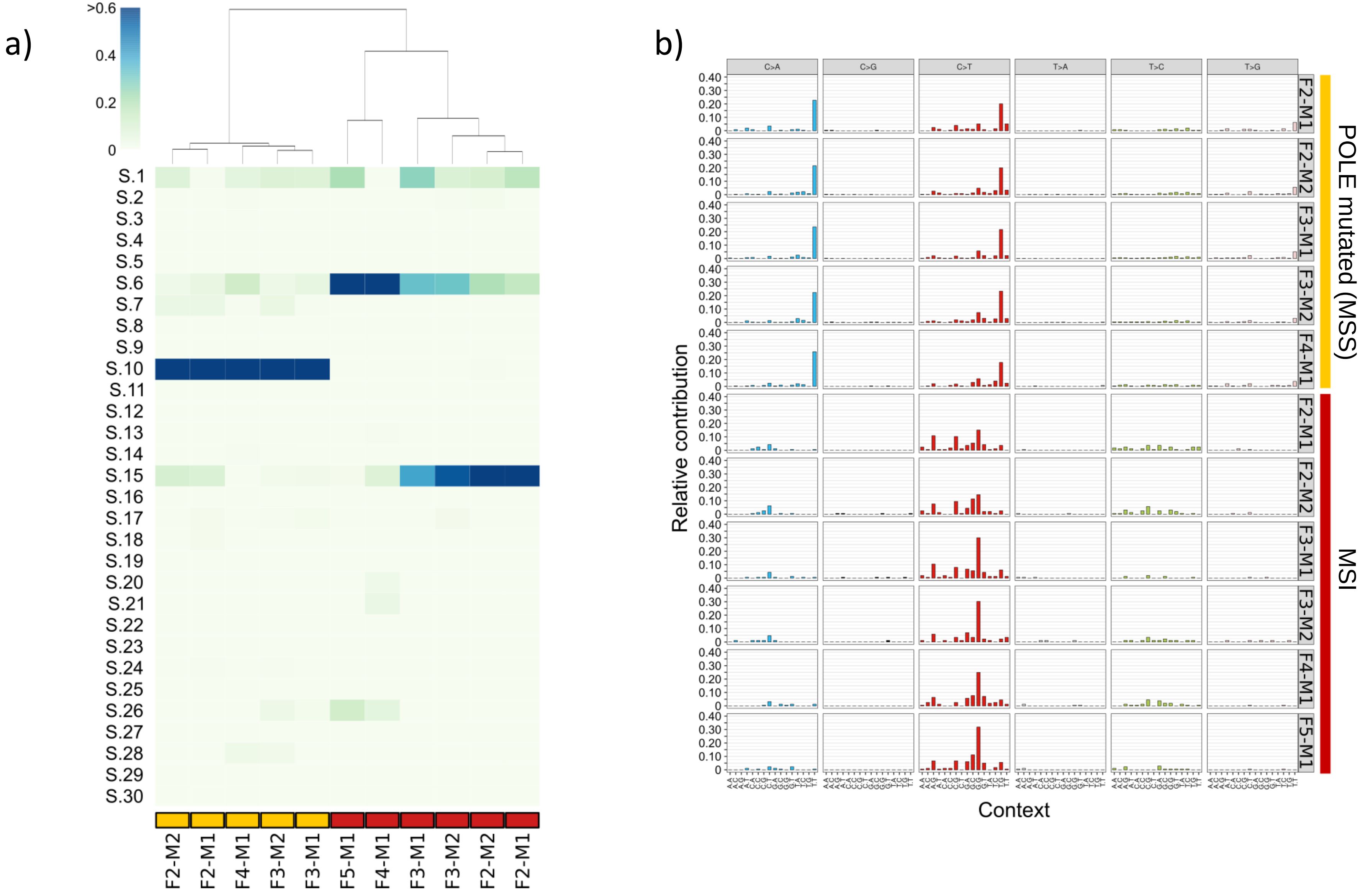

Fig. S13 Mutational signatures acquired during propagation of patient-derived xenografts. Contributions of 30 cancer-associated signatures and signature profiles across evolving PDXs. Genomic variants of the individual PDXs were compared to the corresponding previous generation to infer signatures contributions. a) Clustered heatmap of signature contributions during evolution of the indicated PDX generations. b) Signature profiles acquired in each PDX generation using the six substitution subtypes: C>A, C>G, C>T, T>A, T>C, and T>G. Alterations of each sample were inferred comparing two consecutive generations (see Method for detailed information).

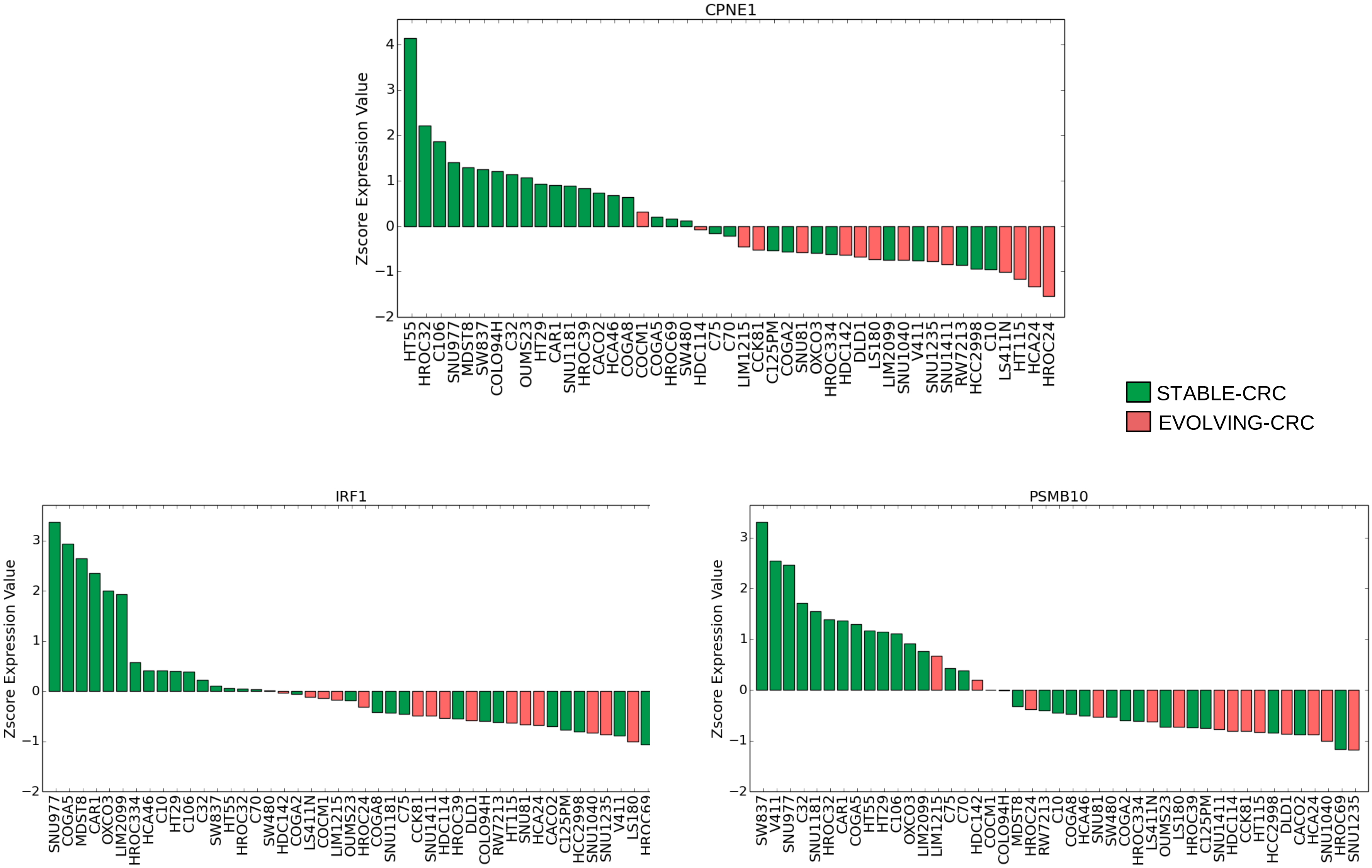

Fig. S14 Gene differentially expressed in EVOLVING-CRC. Waterfall charts show the z-score expression values of IRF1, CPNE1 and PSMB10 across a panel of 45 CRC cell lines.

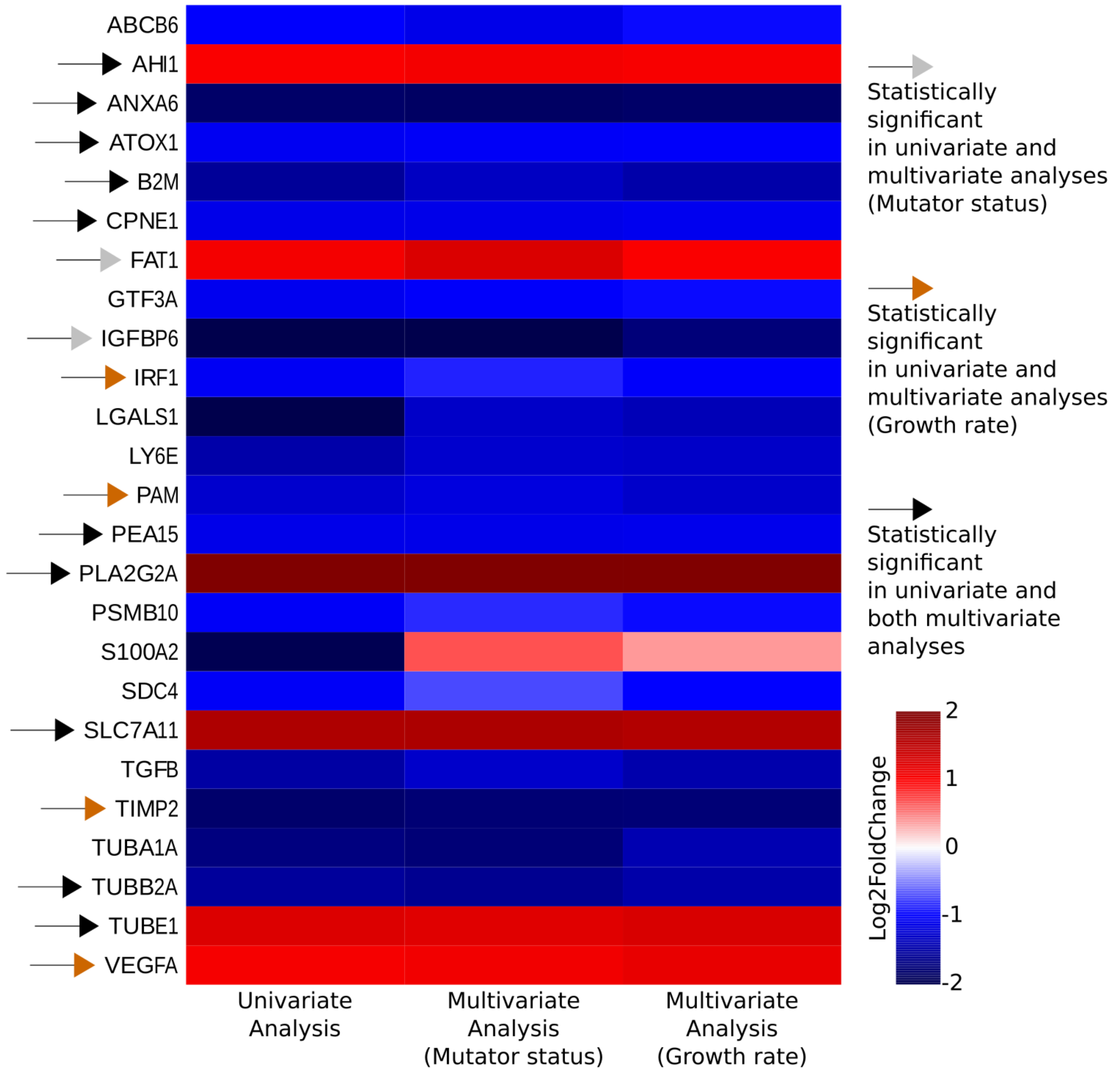

Fig. S15 Gene differentially expressed in EVOLVING-CRC in univariate and multivariate analyses. Log2 fold-change of the genes listed in Fig 9a according to univariate and multivariate analyses considering the mutator status or the growth rates of the cells.
